# A Mobile Self-Assessment and Referral Platform for Family Caregivers of Individuals With Alzheimer Disease and Related Dementias: Protocol for a Pilot Randomized Controlled Trial

**DOI:** 10.2196/90244

**Published:** 2026-04-01

**Authors:** Francesca Falzarano, Annabelle Greenfield, Hannah Mason, Katerina Bumbalova, Elizabeth Rojas, Aleksandar Bumbalov

**Affiliations:** 1 Leonard Davis School of Gerontology, University of Southern California, 3715 McClintock Ave, Los Angeles, CA, United States, 1 914-960-6998

**Keywords:** Alzheimer disease and related dementias, dementia, dementia family caregiving, technology, behavioral intervention

## Abstract

**Background:**

Family caregiving for individuals with Alzheimer disease and related dementias (ADRD) is characterized by increasing complexity, intensity, and demand across the disease trajectory. Formal home- and community-based services can provide knowledge, skills, and resources to enhance preparedness and self-efficacy, which may protect against adverse caregiving outcomes; however, awareness and uptake of these services remain low. As caregivers increasingly turn to the internet for information and support in their role, technology offers an opportunity to create a more seamless pipeline between assessment and service referral to match family caregivers with targeted services that meet their specific needs.

**Objective:**

The primary objective of this study is to evaluate the feasibility and acceptability of CarePair—a mobile self-assessment and service referral platform—among ADRD family caregivers. Secondary objectives are to assess the preliminary efficacy of CarePair in reducing stress, depressive symptoms, and anxiety, and enhancing self-efficacy among caregivers randomized to the intervention versus an attention control condition. This study also aims to generate preliminary effect size estimates to inform sample size calculations for a future fully powered randomized controlled trial (RCT).

**Methods:**

This pilot RCT will evaluate the feasibility, acceptability, and preliminary efficacy of CarePair. Eighty ADRD family caregivers will be enrolled and randomized in a 1:1 ratio to the intervention (n=40) or an attention control condition (n=40). Recruitment will be facilitated by the project study site located in an urban metropolitan area of the United States, targeting participants who report residing in and/or being in close proximity to any of the following locations: New York City, Long Island, and Westchester County, New York; Seattle, Washington; and Los Angeles, California. Primary feasibility outcomes include recruitment, retention, and completion rates; website usability; and intervention satisfaction. Exploratory analyses will assess preliminary efficacy on stress, depressive and anxiety symptoms, and self-efficacy.

**Results:**

This trial was funded by the National Institute on Aging in September 2023 and received approval from the institutional review board of the University of Southern California on September 10, 2025. Recruitment began in September 2025 and is scheduled to conclude in May 2026, with data collection scheduled to end in August 2026. As of February 2026, 44 participants have been enrolled and 22 have completed the study.

**Conclusions:**

This pilot trial will offer foundational evidence regarding the feasibility and acceptability of the CarePair intervention. Study findings will determine if “go” criteria are met to warrant the advancement to a larger-scale efficacy trial. Participant insights will also be used to guide intervention refinements and digital platform optimization. By offering a low-burden, caregiver-centered mobile app, CarePair has the potential to facilitate and streamline the timely identification of needs and referral to relevant services for ADRD family caregivers.

## Introduction

### Background

Approximately 7.2 million individuals have Alzheimer disease and related dementias (ADRD) in the United States, and 83% of these individuals are reliant on over 12 million unpaid family members and/or friends for basic assistance [1]. While framed as a policy solution to ease strain on the health care system, the lack of viable alternatives for long-term care makes family caregiving unavoidable to meet the needs of the growing ADRD population [2]. Despite their crucial role, the significant physical, psychological, and economic challenges associated with ADRD caregiving significantly increase the risk for a myriad of poor outcomes, including caregiver burden, depression, anxiety, and poor quality of life [3-5]. The unique nature of ADRD requires caregivers to assume increasingly complex and unpredictable responsibilities with disease progression, often learning “on the fly” with little knowledge or preparedness. Such unmet needs also increase the risk for negative care recipient outcomes, including compromised care, poorer well-being, and early long-term care placement [6,7].

Substantial research has examined the caregiving experience to elucidate how family caregivers can be better supported to effectively provide care and to circumvent or delay long-term care placement of the care recipient [7,8]. Findings in this arena have informed numerous intervention efforts aimed at mitigating care-related stress and promoting well-being [9]. For example, multicomponent interventions targeting training, education, and support can improve caregiver outcomes by providing skills to effectively manage care, albeit with small-to-moderate effect sizes [10]. While evidence-based behavioral interventions show promise for improving ADRD caregiver outcomes, the implementation and scalability of such programs in real-world settings remain limited [11]. A major barrier hampering such efforts is the sheer heterogeneity in the family caregiving experience and trajectory, as well as differential outcomes resulting from care-related stress [12]. This variability can limit the effectiveness of interventions designed to alleviate caregiver burden, as a “one-size-fits-all” approach is insufficient for meeting the diverse needs of ADRD family caregivers.

### Theoretical Framework

The stress process model [13] provides a theoretical framework to elucidate the dynamic nature of the caregiving experience and the ways by which care-related stress—and appraisals of such stress—differentially influence caregiver outcomes. In addition to background and contextual characteristics, the model is divided into four domains: (1) primary stressors, which are derived directly from the care environment (eg, care recipient’s functional and cognitive status); (2) secondary stressors, in which caregiving responsibilities spill over into other life domains (eg, interference with work, family conflicts, and role strains); (3) mediators (eg, support); and (4) outcomes (eg, well-being). The dynamic interplay among these various dimensions of stress differentially shapes caregiver outcomes [14,15]. While prior work underscores the importance of addressing secondary stressors to protect against poor outcomes, they are rarely considered, assessed, or addressed in real-world clinical or community settings [13,16,17].

Further, the model posits that mediating factors, including external (eg, social support) and internal (eg, adaptive coping) factors, can influence the appraisal of care-related stress and subsequently alleviate some of the impact of caregiving stressors. Formal services and supports, such as education, respite, and personal care, can offer knowledge and skill-building opportunities that enhance resilience and adaptive coping. This, in turn, bolsters caregiving self-efficacy, preparedness, and positive caregiving appraisals, thereby promoting quality of care of the care recipient and quality of life among the care dyad [18].

### Home- and Community-Based Services

While formal services may enhance caregiver support, actual utilization remains strikingly low in real-world settings. Utilization rates are estimated at 26% and 28% for home care and adult day services, respectively, with home care being the only required home- and community-based service (HCBS) for states participating in Medicaid [19-21]. Findings regarding the uptake of respite (12.7%), skills training (6.3%), and support groups (<5%) further highlight significant underutilization [22]. Barriers contributing to this low prevalence include geographical, occupational, cost, and knowledge-related constraints; cultural incongruence; and uncertainty about services, in addition to information overload by placing the burden on caregivers to seek support [23]. These issues are further compounded by the lack of protocols in most health and long-term care systems for effectively recognizing and integrating caregivers into care delivery processes or for systematically assessing the heterogeneity in caregivers’ needs, preferences, and barriers [24].

Our previous work has identified cross-dimensional barriers that impede the identification, access, and actual utilization of formal services and supports [25]. These gaps—described as “nonstarters” to HCBS—exacerbate both primary and secondary stressors, thereby amplifying the likelihood of greater stress and burden. From a structural lens, the siloed, fragmented nature of the service system and the complex bureaucratic structure in which it is embedded contain unclear entry points, which can overall serve as a major deterrent from seeking support, causing those most in need of support to “fall through the cracks” [26]. Despite this pattern, caregivers frequently cite the need for services targeting educational, emotional, and informational support, as well as guidance on navigating the health care system.

Recommendations set forth by The National Academies of Science, Engineering, and Medicine emphasize the need for systematic identification, assessment, and referral to enhance relevant supports to sustain their ability to provide high-quality care [27]. Despite this emphasis, issues related to assessment further impede continuity in the pipeline to HCBS utilization. While there are no shortages of measurement tools to capture caregiver burden, some of which are deemed the “gold standard,” from a psychometric perspective, with few exceptions [28,29], these instruments have not been designed to elicit information that can be used to inform appropriate, actionable referrals [24]. Assessments are further limited by a narrow focus on burden as a single construct and by the time required for clinical administration. In order to achieve its intended purpose in real-world settings, assessments require a holistic understanding of caregiving challenges across a wide range of dimensions such as types of tasks, stress and well-being, and types of training and support needed [22,30]. Effective assessments that account for the multidimensionality of ADRD family caregiving could then be used to facilitate targets for referral to relevant formal services and supports [31].

### Technology to Bolster Caregiving Support

While caregivers have expressed a need for personalized guidance, such as formal points of contact, technology is also recognized as a valuable tool with the potential to transform care management, enhancing critical aspects of care such as health monitoring, safety, and communication. As technology becomes increasingly ubiquitous in daily life, it also overcomes barriers to health-related information dissemination to populations previously deemed “hard to reach” [32]. Moreover, technology can support often overlooked aspects of caregiving, including role adaptation, stress reduction, preparedness, and improvement of the overall quality of life and well-being of both caregivers and care recipients [33,34]. As caregivers increasingly turn to the internet for support [25], the potential for digital tools to enhance personalized caregiving support is a promising and cost-effective means to augment support [35,36]. Innovations in technology can facilitate the creation of a more seamless pipeline between caregiver assessment and service referral, thus increasing the potential to bring a “one-stop service” for ADRD caregiving needs to fruition. Further, while the development and implementation of caregiver-focused digital technologies are booming in private industry, research, and clinical settings, the biggest limitation remains the lack of inclusion of family caregivers as co-design partners to ensure that tools are useful, usable, relevant, and aligned with their needs and preferences, which can be considered a major impediment to user adoption and thus scalability. User-centered design approaches are critical for caregiving technology-based interventions to be useful and usable for caregivers, as well as for streamlining pathways to more easily find, access, and utilize formal services.

Among the burgeoning, evidence-based research, tailored support initiatives have emerged; however, many of these initiatives are limited by reliance on an interventionist and a lack of pre-emptive consideration of service use barriers (eg, availability and cost), background characteristics (eg, kinship), and preferences (eg, timing and modality) before referral, which can limit scalability [24,37,38]. Research evaluating the effectiveness of technology-based programs on caregiver outcomes likewise remains limited. Specifically, recent systematic reviews and meta-analyses synthesizing evidence on technology-based behavioral interventions for ADRD caregivers have reported small reductions in depression, anxiety, and caregiver burden, along with modest increases in self-efficacy and quality of life [39-41]. Notably, 1 review found larger effect sizes for mobile-based apps compared to web-based interventions (Hedges *g*=−0.28) [39]. This pattern is unsurprising, given estimates from the Pew Research Center indicating that 98% of Americans own a smartphone, with high and growing adoption among older adults (90% among those aged 50‐64 years and 78% among those aged 65 years or older) [42].

At the same time, these reviews highlight important limitations in the existing literature, with most studies categorized as having low-to-moderate quality. Substantial variability in intervention content and delivery, along with inconsistent methodologies, outcome measures, and reported effects, further complicates ascertaining the mechanisms underlying intervention effects, which limits the ability to draw clear conclusions about effectiveness [39,43]. Nonetheless, this limited evidence suggests that multicomponent interventions are most promising, particularly those offering some combination of components, such as training, education, professional support, and relaxation strategies, with interventions that include a personalization component yielding the strongest results [41,43]. Despite the abundance of emerging caregiving-focused digital solutions across both research and industry, few approaches, if any, integrate psychological theory with user-centered design principles to guide both technology and intervention development and delivery in order to combine assessment with referral. Such interdisciplinary approaches are critical for ensuring usability and acceptability and for engaging key stakeholders throughout the development process, all of which are key predictors of adoption.

### Our Study

Given the need for systematic identification, assessment, and referral to ensure that caregivers have the skills and preparedness required to sustain high-quality care and considering the gaps in existing research and intervention efforts, the purpose of this study is to examine the feasibility, acceptability, and preliminary efficacy of technology-facilitated caregiver assessment and referral. CarePair is a mobile app consisting of a personalized self-assessment and service referral platform for ADRD family caregivers that applies machine learning algorithms to match caregivers’ needs with relevant services and supports while proactively accounting for barriers (eg, cost and modality) and individual preferences (eg, timing and distance). By integrating theory, user-centered design, and principles of psychological science, this study aims to evaluate the potential of this novel, integrative approach to improve well-being and quality of life among a diverse population of ADRD family caregivers.

### Aims and Objectives

Our overarching aim is to outline the protocol for a pilot randomized controlled trial (RCT) of the CarePair intervention for ADRD family caregivers. CarePair is designed to enhance awareness and utilization of formal services and supports by directing caregivers to resources that align with their current needs, with the overall goal of promoting caregiver mental health and well-being.

#### Primary Objective

The primary objective is to evaluate the feasibility and acceptability of the CarePair intervention among ADRD caregivers.

#### Secondary Objective

The secondary objective is to assess the preliminary efficacy of CarePair in reducing stress, reducing depressive and anxiety symptoms, and enhancing self-efficacy among ADRD family caregivers.

#### Exploratory Objective

The exploratory objective is to generate preliminary effect size estimates to inform sample size calculations for a future fully powered RCT.

## Methods

### Study Design

This protocol paper follows the SPIRIT (Standard Protocol Items: Recommendations for Interventional Trials) reporting guidelines for pilot protocols. The CarePair SPIRIT checklist is presented in Checklist 1.

This single-site, unblinded pilot RCT will evaluate the feasibility, acceptability, and preliminary efficacy of the CarePair intervention. Eighty ADRD family caregivers will be recruited and randomly assigned to either the intervention group (CarePair) or an attention control group and will participate for a duration of 6 weeks. Intervention participants will receive access to the CarePair app, while control participants will receive access to a digital folder of caregiving articles via the Digify online platform (described below).

### Study Setting

Study activities will be conducted virtually and led by researchers at the University of Southern California (USC) in Los Angeles, California. All study activities, including informed consent, intervention delivery, and outcome assessment, will be conducted virtually. Data will be collected and managed using Research Electronic Data Capture (REDCap), a secure, Health Insurance Portability and Accountability Act (HIPAA) compliant electronic data management system hosted at USC [44,45]. Assessments will be administered at baseline and follow-up at weeks 2, 4, and 6 (postintervention). Intervention group participants will also be invited to complete an optional semistructured interview via Zoom (Zoom Communications) to provide feedback about their experiences and provide suggestions for improvement.

### Eligibility

#### Inclusion Criteria

To participate, individuals must (1) be the primary caregiver of a community-dwelling family member or friend with ADRD; (2) provide at least 10 hours of care per week for at least 6 months; (3) be 18 years of age or older; (4) reside in and/or report being in close proximity to any of the following locations: New York City, Long Island, and Westchester County, New York; Seattle, Washington; and Los Angeles, California; (5) have regular internet access; (6) have regular access to a smartphone and proficiency using a smartphone; (7) speak English fluently; (8) not report cognitive impairments (assessed via self-report); and (9) be willing and able to provide informed consent.

#### Exclusion Criteria

Individuals will be excluded if they (1) are not the primary caregiver of a community-dwelling person with ADRD (eg, bereaved or care recipient resides in memory care); (2) provided care for less than 6 months and/or for fewer than 10 hours per week; (3) are under 18 years of age; (4) do not reside in and/or self-report being near one of the following locations: New York City, Long Island, and Westchester County, New York; Seattle, Washington; and Los Angeles, California; (5) lack regular internet access; (6) lack a smartphone and/or are unable to operate it; (7) are not fluent in English; (8) self-report cognitive impairments; and (9) are unable or unwilling to provide informed consent.

### Sample Size

This study proposes to enroll 80 ADRD family caregivers, randomized in equal numbers (n=40 per group) to either the intervention or attention control conditions. Consistent with stages 0-1B of the National Institutes of Health (NIH) stage model for behavioral intervention development [46], the primary goal of this pilot study is to establish the feasibility and acceptability of CarePair. Accordingly, the study is not powered to detect clinically meaningful differences in outcomes. A sample size of 40 participants per arm exceeds the upper limit recommended (n=35) for feasibility trials [43] and allows for adequate precision in estimating key feasibility parameters (recruitment, retention/attrition, and platform usability), which will inform prespecified progression (go/no-go) criteria for advancing to a full-scale RCT. This approach is consistent with methodological guidance for pilot and feasibility studies, which prioritizes the estimation of feasibility parameters over formal hypothesis testing or power-based sample size calculations [47-50]. Preliminary efficacy will be explored as a secondary aim to estimate effect sizes and associated CIs to guide the design of a subsequent, fully powered efficacy trial.

### Intervention Description

This study evaluates technology-facilitated ADRD caregiver assessment and referral. CarePair is a mobile app comprised of a self-assessment and service referral platform designed to support ADRD family caregivers by connecting them with formal services and resources tailored to their unique needs and preferences. Upon enrollment, caregivers will complete a brief 12-item survey that evaluates unmet needs across various care-related domains (eg, mental health, legal guidance, and social support). Responses are used to algorithmically match caregivers with content-relevant resources (eg, blogs and educational materials), as well as community services offered both virtually and in-person (eg, support groups, respite care, and financial education) using Zip Code application programming interface functionality to tailor location-based recommendations according to their reported assessment scores and indicated preferences (eg, cost and service timing).

CarePair’s design and development followed a user-centered design framework to ensure the intervention reflected the priorities, experiences, and feedback of ADRD caregivers. Prior to the commencement of technology development, a stepwise, mixed-methods approach was utilized to identify caregivers’ most pressing service-related needs. In study 1, conducted from June 2022 to August 2023, 63 participants completed a survey battery of caregiving measures assessing stress and psychosocial well-being to elucidate the most common unmet needs reported by ADRD caregivers. A subgroup of this sample (n=34) participated in one-on-one semistructured interviews exploring their caregiving experiences, including discussion of any family support, the diagnosis process, their current needs, and the utilization of HCBSs, as well as the potential barriers and facilitators of HCBSs. In study 2, data gathered during study 1 activities were used to inform 5 focus group sessions comprised of ADRD family caregivers (n=13) and subject matter experts (SMEs; n=17; eg, care consultants and dementia trainers), which were conducted in April 2023 to gather a holistic, multiperspective understanding of the major barriers and facilitators of service use, as well as potential solutions [25].

Data collected in the preceding fact-finding phases of the study informed the content, design, and functionality of an initial CarePair prototype. Version 1.0 of the CarePair mobile app underwent several rounds of refinement to enhance usability and accessibility based on feedback from the project team. Following internal review, semistructured interviews were conducted (25 participants, 15 caregivers, and 10 SMEs) from August to October 2024 to gather comprehensive feedback related to the app’s content, design, usability, and functionality, which informed further refinements to the platform. This stakeholder-informed development and iterative-feedback process helped ensure that the intervention was contextually relevant, acceptable, and usable prior to launching the RCT phase of the project.

### Study Conditions

Participants will be randomly assigned to the intervention group or the attention control (Digify) group. Both groups will log into their assigned platform (CarePair or Digify) twice per week and read 1 article per log-in (12 articles total across the intervention) for the 6-week study period. Participants will access their respective platform via their personal smartphone device.

### Intervention Group

Participants assigned to the intervention group will have access to the CarePair app. Over the 6-week study period, they will be instructed to log in twice per week to read and engage with 1 article per log-in session (eg, by rating, commenting, saving, or taking notes). Participants will also be encouraged, but not required, to explore and engage with service referrals available through the platform. Backend data will be collected to measure app engagement, including participants’ number of log-ins, time spent engaging with materials, specific content accessed, and feature interactions.

The onboarding process will direct caregivers to create a profile for themselves and their care recipients, choose an avatar, and select service preferences (eg, modality, cost, distance based on zip code, and time of the day). Users will also be prompted to complete a 12-item assessment (administered at pre- and postintervention)—the content of which was informed by studies 1 and 2 described above—to measure the primary domains caregivers reported needing assistance with. Each item begins with the stem “In your role as a caregiver, how much support do you need with…” followed by items such as “Understanding your care recipient’s brain change?” and “Dealing with your own feelings and worries?” Response options range from 1 (no need) to 4 (high need).

**Figure 1.**
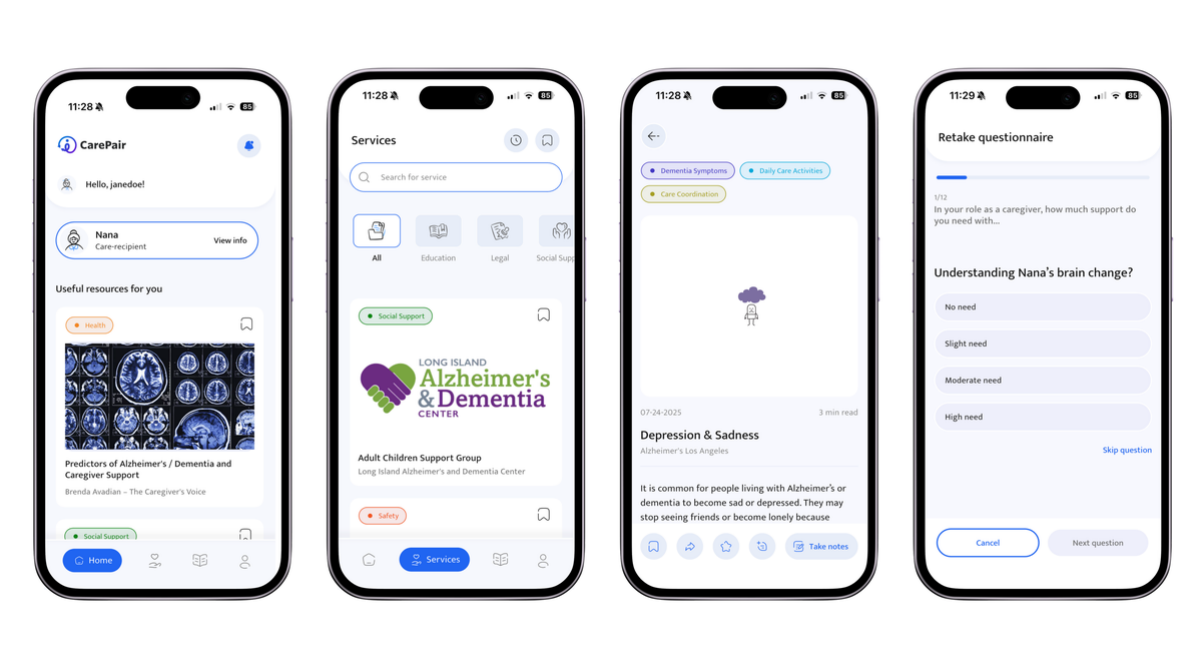
CarePair app interface.

Following the initial onboarding session, users will be provided with an in-app tutorial that introduces the platform’s primary features (see Figure 1 for screenshots of the CarePair app interface). The *Home* page provides a personalized feed of resources curated algorithmically based on the user’s reported needs and preferences. The *Services* tab includes a catalog of local and virtual services tailored to caregivers’ profile preferences and assessment responses gathered during onboarding. The *Library* tab allows participants to browse through the app’s vetted ADRD- and caregiving-related resources, thematically organized by domain and relevance. Both the *Services* and *Library* pages allow users to add filters by category (eg, social support, legal, education, and grief).

Further, caregivers can interact with resources by commenting, rating, saving, sharing, or note-taking directly in the app. Comments and ratings are visible to other caregiver users. To protect confidentiality, users are instructed during onboarding to create pseudonymous usernames and avoid sharing personally identifiable information. Research staff review account registrations and may modify usernames if they contain personally identifiable information. User-generated content is routinely monitored by research staff. Comments will be removed if they include identifiable information, misinformation, promotional content, or potentially harmful or inappropriate language. These procedures are intended to reduce privacy risks and support a safe user environment. Finally, caregivers can edit their profiles and needs assessment responses, manage settings, review notes and saved resources, and access a directory of national ADRD-focused organizations in the *Account* tab.

All information related to services and resources will be maintained by the study team via a secure database repository. Within the app, caregivers may also flag content that is inaccurate, inaccessible, or outdated for review by the investigative team.

### Control Group

Participants in the attention control group will access a digital folder of articles via Digify, a secure document-sharing platform that allows for controlled access of materials and user-engagement tracking on metrics, including log-ins, articles read, and time spent viewing articles. This folder contains most articles available in CarePair, all focused on ADRD and caregiving topics (3 articles with video components were excluded as these formats are not supported by Digify), but they are not organized by relevance or tailored to caregivers’ needs. Control participants follow the same engagement schedule as participants in the intervention group (ie, 2 log-ins per week for 6 weeks; read 1 article per session). Control activities are designed to match the intervention condition in terms of time commitment and interface interaction, and to ensure consistent engagement across groups while allowing for the isolation of the impact of CarePair’s delivery and personalization features. During onboarding, participants are provided with a deidentified username and password to access the Digify platform, along with step-by-step instructions on the use of the platform.

### Recruitment

Sample recruitment will be facilitated by the project’s study site using a multipronged outreach strategy targeting ADRD caregivers in the following locations: New York City, Long Island, and Westchester County, New York; Seattle, Washington; and Los Angeles, California. Recruitment strategies will include: (1) posting study flyers in high-traffic areas in the abovementioned cities; (2) study announcements made through community organizations; (3) ResearchMatch, a NIH-funded online registry that connects research volunteers with institutional review board (IRB)–approved studies; (4) digital outreach, including social media advertisements on platforms (eg, Facebook and Instagram); and (5) outreach to organizations provided as resources within the CarePair app. Additionally, individuals who have participated in prior studies and have provided consent for recontact will be approached to gauge interest and eligibility. Snowball sampling through community networks, social media, and currently enrolled participants will also be encouraged. All interested individuals will be directed to complete the study’s screening survey to determine eligibility.

### Ethical Considerations

This study was approved by USC’s IRB on September 10, 2025 (protocol#: UP-25‐00690). The study IRB approval letter can be found in Multimedia Appendix 1. The trial is registered on ClinicalTrials.gov (NCT06418971). The trial registration record includes all items from the World Health Organization trial registration dataset (Multimedia Appendix 2), including the primary registry name, trial identifying number, key eligibility criteria, target sample size, primary outcomes, secondary outcomes, funding sources, and sponsor details.

In accordance with the International Council for Harmonisation–Good Clinical Practice E6(R3) guidelines and applicable local regulations, trained IRB-approved research assistants (RAs) will obtain electronic informed consent via REDCap’s e-Consent platform after confirming participant eligibility.

During the virtual consent meeting, RAs will review the study purpose, study procedures, and participant rights (eg, the right to withdraw at any time without penalty) to support participant comprehension, and will encourage questions at any time. They will also explain what participation entails, including time commitments, responsibilities, and potential risks. As consent is an ongoing process, participants will be promptly informed of any protocol changes that could affect their willingness to continue. After the consent meeting, participants will be emailed a copy of the informed consent form signed by both themselves and the consenting RA. See Multimedia Appendix 3 for the informed consent form.

All study personnel are trained in human participants’ protection, confidentiality, and recruitment procedures. Data collection, sharing, and storage will comply with HIPAA and IRB regulations. Each participant will be assigned a unique study identifier, and all data will be stored in a secure, password-protected REDCap database accessible only to IRB-authorized personnel. To maintain confidentiality, all datasets will remain deidentified throughout the study, including during analysis and dissemination.

Participants will receive US $25 for each completed survey and US $20 for an optional interview, for a maximum total of US $70. All payments will be sent as Amazon digital gift cards.

### Data Management

All study data will be stored on a secure, password-protected server with multifactor authentication, accessible only to IRB-authorized study personnel. Supported by the Southern California Clinical and Translational Science Institute (CTSI), REDCap [44,45] will be used for participant tracking, survey data collection, and consent procedures. As a secure, web-based data management platform, REDCap enables the development of customized data management and capture systems with online entry forms, reporting tools, automated export procedures for seamless data download to common statistical packages, and procedures for importing data from external sources. This database system is also utilized for the provision of data to the IRB, NIH, and US Food and Drug Administration and includes robust security features, including role-based access controls, Institutional Lightweight Directory Access Protocol and Shibboleth single sign-on (SSO) authentication, and a complete audit trail of data entry and export activities. REDCap is hosted on CTSI-managed servers, which are backed up daily and support encrypted (SSL-based) connections. The platform is maintained nationally through a multi-institutional consortium led by the Vanderbilt University Clinical and Translational Science Award program.

### Randomization Process

After providing consent and completing the baseline survey, participants will be randomized to either the intervention or attention control group using REDCap’s randomization module. A stratified permuted block randomized design is utilized to enhance internal validity and minimize potential confounding variables. Randomization tables are generated by an RA, who is not involved in data collection or participant randomization, using a custom Python script.

To ensure demographic balance across study arms while accommodating the target sample size (n=80), randomization is stratified based on caregiver sex and race/ethnicity, resulting in 24 unique strata. Within each stratum, 25 permuted blocks of 4 are created, each containing 2 “intervention” and 2 “control” assignments, randomly shuffled using Python’s random shuffle() function to ensure unpredictability. The full table is compiled into a .csv file and uploaded to REDCap’s secure randomization module. Once uploaded, the allocation table is locked and inaccessible (ie, no longer visible or modifiable) to study staff. Participants will be randomized in real time via REDCap after stratification variables are entered (sex and race) to preserve allocation concealment and ensure balanced/methodologically sound group assignment. Once randomized, study staff are unable to change participants’ group assignments.

### Assessments and Outcomes

Prospective participants will complete a brief eligibility screening questionnaire in REDCap, which includes basic demographic items (eg, age and sex) and questions aligned with study inclusion/exclusion criteria (eg, “Do you speak English fluently?”). Study assessments will be administered electronically via REDCap at 4 time points: baseline, brief check-in surveys at the end of weeks 2 and 4, and 6-week follow-up. Intervention participants will also be invited to complete an optional postintervention feedback interview via Zoom or telephone.

**Figure 2.**
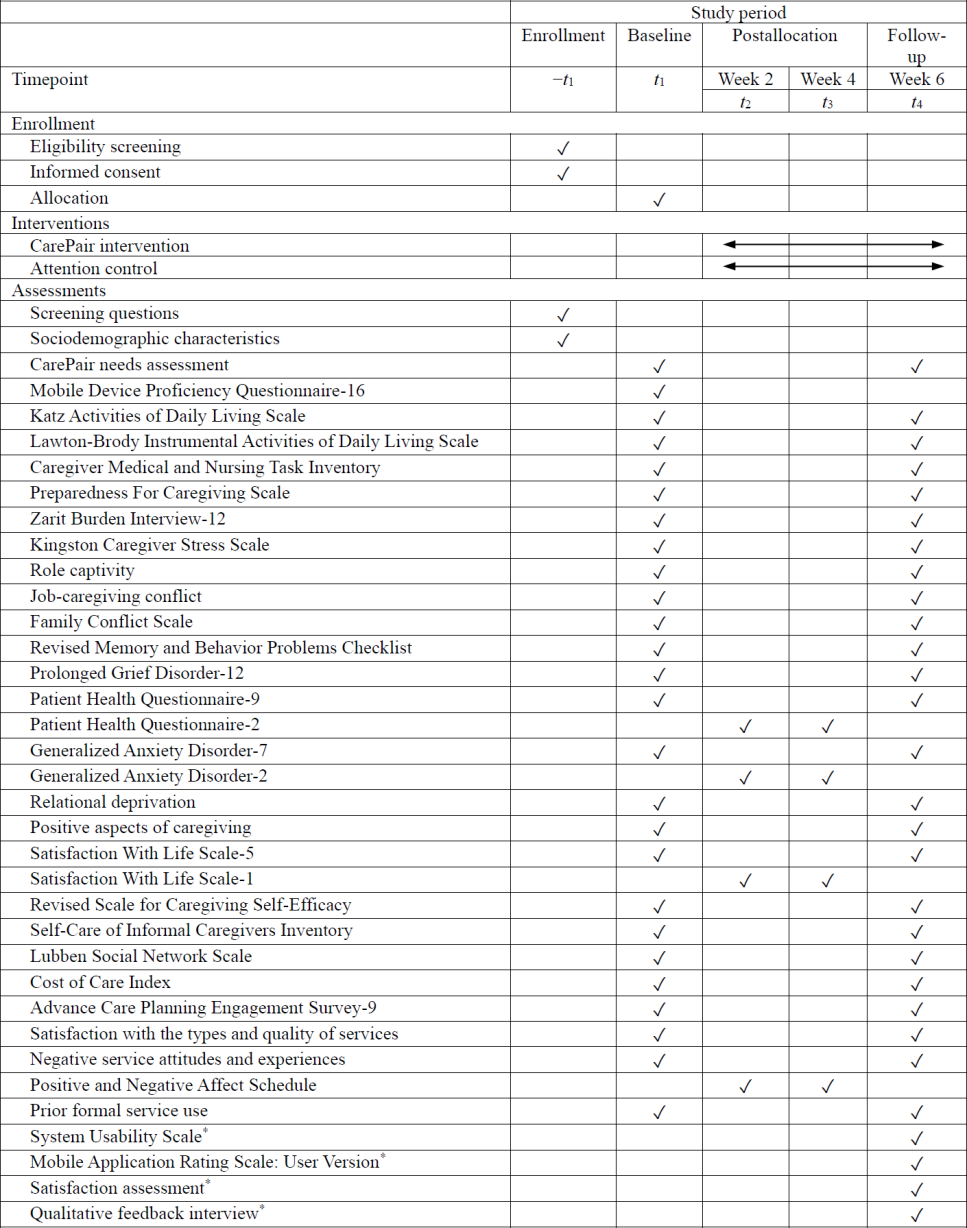
SPIRIT (Standard Protocol Items: Recommendations for Interventional Trials) checklist: CarePair trial assessments and timepoints. *Intervention group only.

A description of study measures, including assessment time points, is shown in Table 1. See Figure 2 for the enrollment and intervention schedule.

**Table 1.** Description of survey instruments based on the stress process model [13].

Domain and measure		Time points			
		S^a^	BL^b^	Weeks 2 and 4	FU^c^
Background and context					
Sociodemographic characteristics					
Single-item assessments (age, sex, race/ethnicity, education, geographic location, income, and kinship to care recipient)		✓	—^d^	—	—
Technology proficiency					
Mobile Device Proficiency Questionnaire-16 [51]		—	✓	—	—
Primary stressors: objective					
Functional dependency					
Katz Activities of Daily Living Scale [52,53]		—	✓	—	✓
Lawton-Brody Instrumental Activities of Daily Living Scale [54]		—	✓	—	✓
Caregiver Medical and Nursing Task Inventory [55,56]		—	✓	—	✓
Cognitive impairment					
Revised Memory and Behavior Problems Checklist [57]		—	✓	—	✓
Primary stressors: subjective					
Relational deprivation					
Relational Deprivation Scale [13]		—	✓	—	✓
Secondary stressors: role and intrapsychic strains					
Family conflict					
Family Conflict Scale [13,58]		—	✓	—	✓
Employment conflict					
Job-Caregiving Conflict Scale [13]		—	✓	—	✓
Mood					
Positive and Negative Affect Schedule [59]		—	—	✓	—
Preparedness					
Preparedness for Caregiving Scale [60]		—	✓	—	✓
Cost of care					
Cost of Care Index–Economic Costs Subscale [61]		—	✓	—	✓
Surrogate decision-making					
Advance Care Planning Engagement Survey-9 [62,63]		—	✓	—	✓
Self-care					
Self-Care of Informal Caregivers Inventory [64]		—	✓	—	✓
Role confusion					
Role Captivity Scale		—	✓	—	✓
Predeath grief					
Prolonged Grief Disorder-12 Caregiver Version [65,66]		—	✓	—	✓
Caregiver burden					
Zarit Burden Interview-12 [67]		—	✓	—	✓
Caregiving rewards					
Positive aspects of caregiving [68]		—	✓	—	✓
Mediators					
Caregiver needs					
CarePair needs assessment^e^		—	✓	—	✓
Social support					
Lubben Social Network Scale-6 [69]		—	✓	—	✓
Formal support					
Negative service attitudes and experiences [70]		—	✓	—	✓
Satisfaction with the types and quality of services [71]		—	✓	—	✓
Prior use of services, willingness to use services, and types of services used^e^		—	✓	—	✓
Primary outcomes					
Feasibility					
Recruitment: number/proportion screened, eligible, consented/enrolled, declined, and retained [72]		—	✓	—	✓
Usability: System Usability Scale (SUS) [73,74]; user version of the Mobile Application Rating Scale (uMARS) [75]		—	—	—	✓
Intervention adherence: app usage (measured via number of log-ins); feature usage (measured via number of interactions with resources [eg, saves and notes])		—	—	—	✓
Acceptability					
Satisfaction and acceptability survey^e^		—	—	—	✓
Secondary outcomes					
Depression					
Patient Health Questionnaire-9 [76]		—	✓	—	✓
Patient Health Questionnaire-2 [76,77]		—	—	✓	—
Anxiety					
Generalized Anxiety Disorder-7 [78]		—	✓	—	✓
Generalized Anxiety Disorder-2 [78]		—	—	✓	—
Caregiver stress					
Kingston Caregiver Stress Scale [79,80]		—	✓	—	✓
Self-efficacy					
Revised Scale for Caregiving Self-Efficacy [81]		—	✓	—	✓
Quality of life					
Satisfaction with Life Scale-5 [82]		—	✓	—	✓
Satisfaction with Life Scale-1 [82,83]		—	—	✓	—

a S: screening survey.

b BL: baseline survey.

c FU: 6-week follow-up survey.

d Not applicable.

e Developed for this study.

#### Primary Outcomes

##### Feasibility

Feasibility will be evaluated across recruitment, retention, and app usability. *Recruitment feasibility* [72] will be assessed by tracking the number and proportion of caregivers who (1) are eligible and consent, (2) are eligible and decline participation, and (3) are screened and found eligible. These metrics will be recorded in REDCap using timestamped research logs and will be used to calculate consent rates (eg, the number of consented individuals divided by the number of interested individuals). *Retention* will be examined via completion and attrition rates for the full sample and by study arm. We will calculate the number of completed assessments per participant divided by the total number of assessments, with attrition rates calculated as the proportion of noncompleters among enrolled participants. A designated data manager will oversee the collection, monitoring, and reporting of feasibility data.

*App feasibility* will be assessed through backend usage analytics (eg, number of log-ins and in-app interactions such as saving or reviewing resources). To assess *usability*, participants will complete a 10-item modified version of the System Usability Scale (SUS) [73,74], which measures usefulness, ease of use, and satisfaction at the posttest phase. Sample items include “I thought the CarePair app was easy to use” and “I felt very confident using the CarePair app.” Each item is rated on a 5-point Likert scale (1 [strongly disagree] to 5 [strongly agree]). Total scores are converted to a range from 0 to 100, with higher values indicating greater usability. At follow-up, participants will complete the 20-item user version of the Mobile Application Rating Scale (uMARS) [75], which evaluates app quality across 5 domains: engagement, functionality, esthetics, information, and subjective quality. Items such as “Is the app content (visuals, language, and design) appropriate for the target audience?” are rated on a 5-point Likert scale (1 [inadequate] to 5 [excellent]), with higher scores indicating higher perceived quality. These metrics will inform feasibility across recruitment, engagement, retention, and app usability, as well as guide modifications for a future large-scale trial.

##### Acceptability

Acceptability will be assessed at the 6-week follow-up using an 8-item survey measuring user satisfaction, usefulness, and ease of use. Participants will rate their agreement with statements such as “I was satisfied with the features in the CarePair application” and “Using the CarePair application was worth my time” on a 5-point Likert scale (1 [strongly disagree] to 5 [strongly agree]). In addition, CarePair intervention participants will complete 4 open-ended questions providing qualitative feedback on their experiences, including which features were engaging, helpful, or in need of improvement. Sample prompts include the following: “Which CarePair features did you find most valuable? Why?”

### Secondary Outcomes

#### Caregivers’ Needs

Caregivers’ needs will be assessed and compared at baseline and follow-up using a 12-item needs assessment developed for this study. The CarePair Needs Assessment measures caregivers’ needs across a variety of caregiving domains, such as social support, legal guidance, and education. Questions, including “In your role as a caregiver, how much support do you need with connecting with others who are in similar care situations?” are rated from 1 (no need) to 4 (high need). Scores range from 12 to 48, with higher scores indicating greater need.

#### Self-Efficacy

Caregiving self-efficacy will be measured at baseline and follow-up using the 15-item Revised Scale for Caregiving Self-Efficacy [81]. Items assess caregivers’ confidence in managing their own lives in addition to care-related responsibilities (eg, “How confident are you that you can ask a friend/family member to stay with [care recipient] for a week when you need time for yourself?”). Responses are rated from 0 (cannot do at all) to 100 (certainly can do). Total scores range from 0 to 100, with higher scores indicating greater perceived self-efficacy (Cronbach α=0.80).

#### Anxiety

Anxiety symptoms will be assessed using the Generalized Anxiety Disorder-7 (GAD-7) [78]. Participants report how frequently they experienced symptoms (eg, worrying and difficulty relaxing) in the past 2 weeks (eg, “Over the last 2 weeks, how often have you been bothered by... feeling nervous, anxious, or on edge?”). Items are rated on a 4-point scale (0 [not at all] to 3 [nearly every day]). Total scores range from 0 to 21, with higher scores indicating greater anxiety (Cronbach α=0.92).

#### Depression

Depressive symptoms will be measured using the Patient Health Questionnaire-9 (PHQ-9) [76]. Items capture the severity of symptoms (eg, low interest and fatigue) over the past 2 weeks (eg, “Over the last 2 weeks, how often have you been bothered by... feeling tired or having little energy?”). Responses are rated on a 4-point scale (0 [not at all] to 3 [nearly every day]). Total scores range from 0 to 27, with higher scores indicating greater depressive symptoms (Cronbach α=0.89).

#### Stress

Caregiver stress will be evaluated using the 10-item Kingston Caregiver Stress Scale (KCSS) [79,80]. Items measure care-related stress (eg, “To what extent do you have concerns regarding the future care needs of [care recipient]?”), with ratings on a 5-point Likert scale (1 [no stress] to 5 [extreme stress]). Total scores range from 20 to 50, with higher scores reflecting greater caregiver stress (Cronbach α=0.85).

#### Quality of Life

Subjective quality of life will be assessed using the 5-item Satisfaction With Life Scale (SWLS) [82]. Items ask participants to rate agreement with statements, such as “In most ways, my life is close to my ideal,” on a 7-point scale (1 [strongly disagree] to 7 [strongly agree]). Scores range from 5 to 35, with 20 representing a neutral midpoint and higher scores indicating greater life satisfaction (Cronbach α=0.87).

### Retention and Adherence

A structured, multipronged retention and adherence protocol will be implemented at enrollment to promote engagement, minimize loss to follow-up, and ensure complete data collection. Research staff will be trained to respond sensitively to participant needs, including addressing technical issues, emotional strain, and other barriers to engagement.

To reduce attrition and enhance adherence, participants will receive alternating end-of-week emails from their designated RAs, including check-ins at weeks 1, 3, and 5, and brief check-in surveys at weeks 2 and 4. Engagement will be monitored using CarePair usage analytics (eg, log-in frequency and feature usage), which will guide the content of check-in emails. Participants who have logged in will be contacted to assess their experience, address concerns, and offer optional one-on-one support via Zoom or telephone. Those with little or no engagement will receive reminder emails with encouragement and instructions to participate.

Consistent follow-up will be conducted for participants who do not complete the required study activities (eg, surveys and consent). Weekly reminder emails will be sent in the first 3 weeks of no action, followed by a final phone reminder in the fourth week. Participants who remain unresponsive will be considered lost to follow-up. This structured approach is designed to reinforce expectations, identify participation barriers, and provide personalized support to facilitate continued participation.

Retention will also be supported by incentives. Participants will receive a US $25 Amazon gift card after completing the baseline survey and another US $25 gift card after the 6-week follow-up (after completing all required activities). The fully virtual format further reduces barriers, such as transportation and caregiving responsibilities, making participation more accessible.

Control group activities are designed to match with intervention group activities in terms of interaction frequency and engagement level, minimizing the risk of differential dropout between conditions. All instances of nonadherence or withdrawal, along with reported reasons, will be systematically documented in REDCap to inform feasibility and guide refinements for future trials. While attrition rates of 15% to 30% are common in pilot RCTs and often higher in online interventions [84,85], the study team will monitor retention metrics regularly and, if needed, adjust communication strategies or provide more support to sustain engagement.

### Intervention Fidelity

A comprehensive Manual of Operating Procedures has been developed to guide all aspects of training, intervention delivery, and data management. Standardized protocols are in place for participant screening, enrollment, tracking, and communication. All research staff are trained using consistent materials on study procedures, and ongoing team meetings are held to address any issues that may arise with regard to data collection, participant engagement, and CarePair app functionality.

Intervention activities and group assignments will be fixed across participants to ensure consistency. All participants will follow a standardized 6-week study schedule with assessments at predetermined time points. Scripted email templates will be used for all participant communications to maintain uniformity and proactively address potential concerns or barriers to engagement.

To standardize onboarding, intervention participants will receive standardized training materials, including a PDF guide and brief instructional videos on how to use CarePair. The CarePair app’s interface, content, and features will be the same across all intervention participants; however, its organization will be tailored by machine learning algorithms to each participant’s needs and preferences.

### Harms

Given the minimal-risk nature of the study, adverse events (AEs)—defined as any unfavorable or unintended experience associated with participation, whether related to the study or not—are expected to be rare. Potential risks include mild emotional discomfort when responding to survey or interview questions, exposure to ADRD-related content, or minor frustration when navigating digital platforms. There is also a minimal risk of breach of confidentiality. Participants may skip any questions or activities that feel uncomfortable or distressing. Study staff are trained to recognize and respond to participant distress and will provide appropriate referrals, including to the USC Family Caregiver Support Center, for additional support and resources as needed.

All AEs, serious adverse events (SAEs), and unanticipated problems, as well as any protocol deviations, will be documented using designated reporting forms and logs. The principal investigator (PI) will be notified of any AE or SAE within 24 hours, and all required reporting to the USC IRB, designated Safety Officer, and National Institute on Aging (NIA) Program Officer will be conducted in accordance with institutional and federal policies.

### Monitoring

#### Data Monitoring Committee

Given the study’s minimal risk, a formal Data Monitoring Committee is not required for this pilot trial. In line with institutional oversight policies, the study team is required to submit an annual continuing review to serve as a postapproval monitoring report to USC’s IRB, which includes updates on study progress, protocol adherence, and any unanticipated issues and/or AEs. This reporting ensures ongoing compliance with ethical and regulatory standards throughout the study period.

#### Trial Monitoring

No interim analyses or stopping guidelines are planned due to the study’s short duration and minimal risk for participants. The study will follow a structured data and safety monitoring plan. The PI will oversee day-to-day study conduct, data integrity, and participant safety, and the study team will continuously monitor trial progress and data collection to ensure protocol fidelity and protection of human subjects. A designated Safety Officer—independent of the investigative team and study site—will serve in an advisory capacity to the PI and NIA Program Officer. This individual will evaluate the overall progress of the trial, including participant recruitment, data quality, adherence to study procedures, and participant safety.

Safety monitoring reports will be generated every 3 months beginning at the onset of the trial. These reports will summarize aggregate data on participant accrual, retention, demographic characteristics, protocol deviations, and adverse or unanticipated events. Reports will also include analyses of data completeness, timeliness, and overall study progress. The Safety Officer will review safety reports quarterly and provide written feedback and recommendations to the PI and NIA Program Officer regarding the continuation, modification, or termination of the study.

### Statistical Analysis

Descriptive statistics (eg, frequencies, means, and SDs) will be used to characterize the study sample across arms. The intervention’s feasibility and acceptability will be evaluated through recruitment and retention rates, reasons for noncompletion, app usage data (eg, number of log-ins and in-app actions), and participant usability and satisfaction assessments. Independent samples *t* tests for continuous variables and chi-square tests for categorical variables will be used to test for baseline group differences in sociodemographic characteristics and primary and secondary outcome measures (eg, stress and self-efficacy). Relationships among continuous measures will be examined using Pearson correlations. Additional analyses will examine the relationships between covariates (eg, age, education, and kinship), and outcome measures will be examined for inclusion in multivariate models.

To examine preliminary efficacy, within- and between-group differences in secondary outcomes will be assessed. Specifically, we will examine (1) mean values at each time point by group, (2) within-group change from baseline to follow-up, (3) between-group differences in change, and (4) standardized effect sizes (eg, Hedges g) with 95% CIs to guide planning for a future efficacy trial. Missing data will be handled via listwise deletion. A *P *value of .05 will be used to indicate significance in all analyses.

### Dissemination Plan

A final report will be prepared for submission to peer-reviewed journals and scientific conferences to support dissemination to stakeholders across the research-practice-community pipeline, as well as to the general public. Participants who have consented to email communication will also receive a lay-language summary of the results (formatted for accessibility). Authorship of all products will be determined by the PI in accordance with journal guidelines and individual contributions. Deidentified participant-level data will be made available upon reasonable request after study completion and publication.

## Results

**Figure 3.**
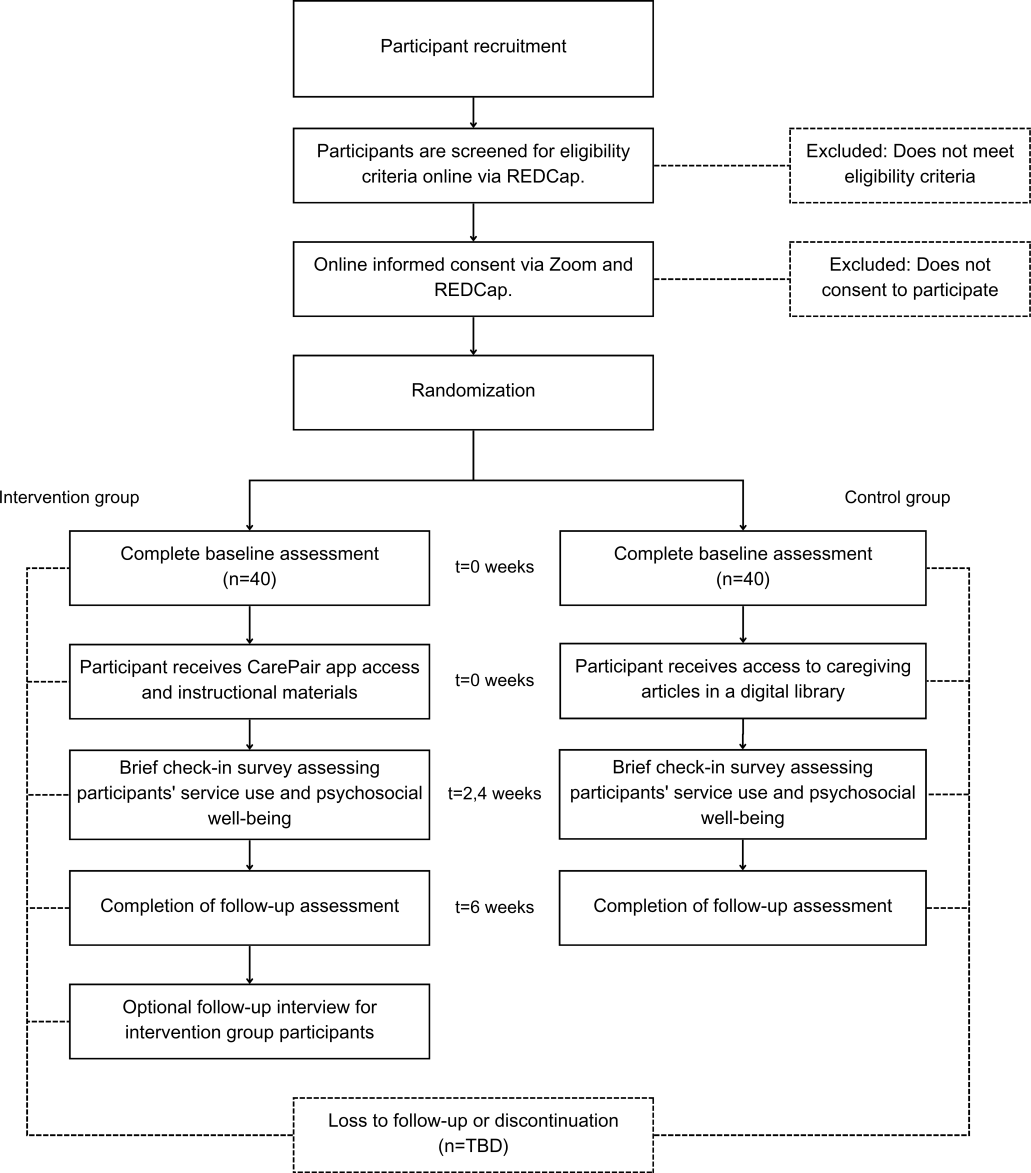
CarePair trial flow diagram. TBD: to be determined.

The trial was funded by the NIA in September 2023 and received IRB approval on September 10, 2025. Recruitment began on September 15, 2025, and is expected to conclude in May 2026, with data collection projected to end in August 2026. As of February 2026, 44 participants have been enrolled and 22 have completed the study. The trial flow diagram is presented in Figure 3. Data analysis is planned for August 2026, and the primary results are anticipated to be available in December 2026.

## Discussion

### Principal Findings

ADRD family caregiving is a chronic, complex role characterized by escalating demands and unpredictability across the disease trajectory and is often assumed with limited knowledge or preparedness. Extensive research has documented the physical and mental health consequences of care-related stress, including increased risks for burden, depression, and anxiety [3-6]. Although the need for systematic identification, assessment, and referral has been emphasized to enhance support, fragmented service systems and navigation barriers contribute to a substantial gap between caregiver needs and the utilization of supportive services [27].

This pilot randomized trial aims to evaluate the feasibility and acceptability of a user-centered, mobile self-assessment and service referral platform for ADRD family caregivers. The current protocol is designed to determine whether CarePair can be feasibly delivered and is perceived as a usable and acceptable digital support tool by family caregivers. We hypothesize that CarePair will (1) demonstrate strong feasibility and acceptability, as reflected by recruitment, retention, adherence, and usability metrics; (2) provide insights on intervention uptake and engagement to inform iterative refinement and future implementation; and (3) generate preliminary effect sizes to guide the design of a fully powered efficacy trial.

Prior behavioral interventions have demonstrated potential to improve caregiver outcomes; however, their implementation in real-world settings remains limited. Although tailored support initiatives have emerged, many rely on interventionist-led models and do not systematically account for service-use barriers, caregiver characteristics, or individual preferences to inform referrals, thereby limiting personalization and real-world applicability [24,37,38]. Studies on technology-based caregiver interventions have reported small reductions in depression, anxiety, and caregiver burden, along with modest improvements in self-efficacy and quality of life [39-41]. However, the overall strength of the evidence remains mixed, with considerable variability in theoretical grounding, personalization, implementation, and demonstrated effectiveness.

CarePair was developed to address these gaps by integrating self-directed assessments with tailored service referrals that also proactively account for barriers (eg, cost and modality) and individual preferences (eg, timing and distance) to streamline ADRD caregivers’ knowledge of and access to relevant support. The design and development of the intervention were guided by the Stress Process Model [13], which conceptualizes caregiving outcomes as arising from the interplay of primary stressors (eg, care demands and functional/cognitive impairment), secondary stressors (eg, role strain and work-family conflict), and mediating factors (eg, social support, coping, and preparedness). A user-centered design framework further informed the platform’s development, emphasizing usability, accessibility, and alignment with ADRD caregivers’ preferences. Together, these approaches strengthen the intervention’s relevance and enhance potential for real-world implementation.

Data on key feasibility indicators, including recruitment, retention, adherence, and participant satisfaction, will determine whether CarePair can be effectively delivered and whether it is perceived as useful and usable among ADRD caregivers. Study findings will guide iterative refinements to CarePair’s content, referral logic, and onboarding processes and will directly inform the design of a larger, multisite RCT to evaluate CarePair’s efficacy for enhancing the mental health and well-being of ADRD family caregivers.

### Limitations

Several limitations warrant consideration. Given that the primary objective of this pilot study is to evaluate feasibility and acceptability, the sample is not statistically powered to examine efficacy for improving caregiver outcomes. Eligibility criteria require participants to have device/internet access, which, in addition to the study’s virtual modes of recruitment, may yield a sample with higher digital literacy than the broader caregiving population. Self-selection bias may also impact generalizability, as individuals who choose to participate may be more motivated, more receptive to digital caregiving tools, or differ in caregiving stress levels, which can bias estimates of feasibility and acceptability. Finally, the relatively short intervention period limits the ability to evaluate sustained engagement, longer-term acceptability, and barriers or facilitators influencing CarePair uptake over time.

### Conclusion

This study will generate foundational evidence on the feasibility, acceptability, and perceived utility of a mobile self-assessment and referral platform to enhance knowledge of and access to formal services and supports for ADRD family caregivers. Findings will inform the design of a fully powered clinical trial and guide the optimization of the platform. As a scalable, low-cost, and low-burden intervention, CarePair has the potential to bridge gaps in the identification-assessment-referral pipeline, with the overall goal of supporting the mental health and well-being of ADRD family caregivers. Future work should examine the influence of CarePair on both caregiver and care recipient outcomes. Future research should also explore how to integrate digital self-assessment and referral systems into existing clinical workflows within home- and community-based organizations. Dissemination of findings will include sharing results with academic, clinical, and community stakeholders to support continued refinement and implementation of digital support solutions for ADRD family caregivers.

## Supplementary material

10.2196/90244 Multimedia Appendix 1 CarePair institutional review board approval letter.

10.2196/90244 Multimedia Appendix 2 World Health Organization trial registration dataset.

10.2196/90244 Multimedia Appendix 3 CarePair study informed consent form.

10.2196/90244 Checklist 1 SPIRIT checklist.

10.2196/90244 Peer Review Report 1 Peer Review Report by the NIA-S - Behavior and Social Science of Aging Review Committee, National Institute on Aging Initial Review Group, National Institute on Aging (National Institutes of Health, USA)

## Abbreviations

ADRD: Alzheimer disease and related dementias
AE: adverse event
CTSI: Clinical and Translational Science Institute
GAD-7: Generalized Anxiety Disorder-7
HCBS: home- and community-based service
HIPAA: Health Insurance Portability and Accountability Act
IRB: institutional review board
KCSS: Kingston Caregiver Stress Scale
NIA: National Institute on Aging
NIH: National Institutes of Health
PHQ-9: Patient Health Questionnaire-9
PI: principal investigator
RA: research assistant
RCT: randomized controlled trial
REDCap: Research Electronic Data Capture
SAE: serious adverse event
SME: subject matter expert
SPIRIT: Standard Protocol Items: Recommendations for Interventional Trials
SUS: System Usability Scale
SWLS: Satisfaction With Life Scale
uMARS: user version of the Mobile Application Rating Scale
USC: University of Southern California
