# Supplementary material for: A Mobile Self-Assessment and Referral Platform for Family Caregivers of Individuals With Alzheimer Disease and Related Dementias: Protocol for a Pilot Randomized Controlled Trial
Source: JMIR Res Protoc. 2026 Apr 1;15:e90244. doi: 10.2196/90244 (PMC13043018; doi:10.2196/90244)
Supplement: Multimedia Appendix 1 [file resprot-v15-e90244-s001.pdf]

University of Southern California Institutional Review Board
3720 S. Flower Street, Suite 325
Los Angeles, CA 90089
Telephone: (323) 442-0114
Fax: (323) 224-8389
Email: hrpp@usc.edu

Date:	Sep 10, 2025, 03:56pm
Action Taken:	**Approve**
Principal Investigator:	Francesca Falzarano, PhD<br>DAVIS SCHOOL OF GERONTOLOGY

Project Title:	**CarePair RCT**
Study ID:	**UP-25-00690**
Funding Types:	Federal Grant/Contract
Details:	Funding Agency: NIA (National Institute on Aging)<br>Contract or Grant Number: n/a<br>PI of Project: Francesca Falzarano, PhD<br>DAVIS SCHOOL OF GERONTOLOGY<br>Title of Project: Pilot Randomized Control Trial of CarePair: An Assessment and Referral Platform to Support Family Caregivers of Alzheimer's Disease and Related Dementias<br>PI of Main Grant: Francesca Falzarano, PhD<br>Title of Main Grant: n/a<br><br>Award ID: ID00001395<br>PI: Francesca Falzarano<br>Award Number: 23-11005-A0001<br>Sponsor: National Institute on Aging

Your submission was reviewed and approved by the University of Southern California Institutional Review Board (IRB) under expedited review (45 CFR 46.111) (6) and (7) on 09-10-25. Continuing review is not required for minimal risk projects in accordance with 45 CFR 46.109(f)(1)(i).

The study team will be contacted 2 years after the approval date in this letter as per the USC HRPP Progress Update. For more information please visit: https://hrpp.usc.edu/hrpp/post-approval-monitoring-pam/#progressupdatefaqs

You are required to ensure that this research and the actions of all project personnel involved in conducting the submission will conform with the research project and its modifications approved by the IRB, IRB Policies and Procedures, and applicable state laws. University of Southern California is in compliance with requirements of human subjects research, including 45 CFR 46, 21 CFR 50 and 56.

Any unanticipated problems involving risks to participants or others, deviations from the approved research, non-compliance, and complaints must be reported to the IRB in accordance with University of Southern California Human Research Protection Program policies and procedures. If this study includes ongoing oversight by a Data Safety Monitoring Board (DSMB) or other such committee, reports generated by the DSMB or oversight committee must be submitted to the IRB.

THE FOLLOWING MATERIALS WERE CONSIDERED FOR REVIEW AND APPROVED:

1. iStar Application dated 08-19-25
2. CarePair RCT Protocol(0.01)
3. CarePair RCT Interview Guide(0.01)
4. CarePair RCT Baseline Survey(0.01)
5. CarePair RCT EMAs(0.01)
6. CarePair RCT Follow-Up Survey(0.01)
7. CarePair RCT Screening Survey(0.01)
8. CarePair RCT Email Scripts(0.01)
9. CarePair RCT Flyers(0.01)
10. CarePair RCT ResearchMatch Contact Message(0.01)
11. CarePair Phase 2 - Information Sheet.docx(0.02)
12. CarePair RCT Informed Consent Form(0.02)

THE FOLLOWING EDITS/MODIFICATIONS WERE MADE TO THE ISTAR APPLICATION BY IRB STAFF:

1. iStar # 24.7 dated information sheet and consent to match date of upload so they are consistent. Both documents are now version-dated 08-19-25.

NOTES:

Thank you for providing the NCT number.

THE FOLLOWING DETERMINATIONS WERE MADE:

Waiver of documentation of Informed Consent 45 CFR 46.117(c) and/or 21 CFR 56.109 (c)(1) for screening (uses information sheet).

Social-behavioral health-related interventions or health-outcome studies must register with **clinicaltrials.gov** or other International Community of Medical Journal Editors (ICMJE) approved registries in order to be published in an ICJME journal. The ICMJE will not accept studies for publication unless the studies are registered prior to enrollment, despite the fact that these studies are not applicable "clinical trials" as defined by the Food and Drug Administration (FDA). For support with registration, go to www.clinicaltrials.gov or contact Kimberly Eudy (eudy@usc.edu).

Approved Documents: view

Important

The principal investigator for this study is responsible for obtaining all necessary approvals before commencing research. Please be sure that you have satisfied applicable requirements, for example conflicts of interest, bio safety, radiation safety, biorepositories, credentialing, data security, sponsor approval, **clinicaltrials.gov** or school approval. IRB approval does not convey approval to commence research in the event that other requirements have not been satisfied.

This is an auto-generated email. Please do not respond directly to this message using the "reply" address. A response sent in this manner cannot be answered. If you have further questions, please contact your IRB Administrator or IRB office.

The contents of this email are confidential and intended for the specified recipients only. If you have received this email in error, please notify istar@usc.edu and delete this message.
