# Supplementary material for: A Mobile Self-Assessment and Referral Platform for Family Caregivers of Individuals With Alzheimer Disease and Related Dementias: Protocol for a Pilot Randomized Controlled Trial
Source: JMIR Res Protoc. 2026 Apr 1;15:e90244. doi: 10.2196/90244 (PMC13043018; doi:10.2196/90244)
Supplement: Multimedia Appendix 2 [file resprot-v15-e90244-s002.docx]

**World Health Organization trial registration dataset**

1. Primary registry and trial identifying number: Clinicaltrials.gov: NCT06418971
2. Date of registration in primary registry: February 27, 2024
3. Secondary identifying numbers (if applicable): NIA K99AG073509; R00AG073509 [U.S. NIH Grant/Contract Award Number]
4. Source(s) of Monetary or Material Support: National Institute on Aging (NIA)
5. Primary Sponsor: University of Southern California
6. Secondary Sponsor(s) (if any): Not applicable
7. Contact for Public Queries: Annabelle Greenfield, B.S., Email: ag69282@usc.edu

Francesca B. Falzarano, Ph.D., Email: falzaran@usc.edu

1. Contact for Scientific Queries: Francesca B. Falzarano, Ph.D., Email: falzaran@usc.edu
2. Public Title: Testing & Refinement of CarePair: An Assessment and Referral Platform to Support Family Caregivers of Alzheimer’s Disease and Related Dementias.
3. Scientific Title: Testing & Refinement of CarePair: An Assessment and Referral Platform to Support Family Caregivers of Alzheimer’s Disease and Related Dementias.
4. Countries of Recruitment: United States
5. Health Condition(s) or Problem(s) Studied: Dementia family caregiver well-being
6. Intervention(s):
   - Experimental: Participants access CarePair, a mobile application-based self-assessment and service referral platform designed to support family caregivers. In-app resources include personalized service referrals (e.g., support groups, respite services) and informational resources (e.g., blogs, educational materials), which are curated using machine learning algorithms that match content to caregivers’ needs based on a 12-item needs assessment and user-entered preferences.
   - Attention Control Condition: Participants access a digital folder containing the same caregiving informational resources available in the CarePair app. However, the materials will not be personalized or organized.
7. Key Inclusion and Exclusion Criteria:
   - Ages eligible for study: ≥18 years
   - Sexes eligible for study: both
   - Accepts healthy volunteers: yes
   - Inclusion criteria: Adults (≥18 years) who are the primary caregiver of a community-dwelling family member or friend with Alzheimer’s disease or related dementias (ADRD); provide at least 10 hours of care per week for ≥6 months; reside in or near New York City, Long Island, or Westchester County, New York; Seattle, Washington; or Los Angeles, California; have regular internet access; have access to and proficiency using a smartphone; fluent in English; do not self-report cognitive impairment; willing and able to provide informed consent.
   - Exclusion criteria: Minors (<18 years); not the primary caregiver of a community-dwelling person with ADRD (e.g., bereaved caregivers or care recipients residing in memory care); provide <10 hours of care per week and/or for <6 months; do not reside in or near New York City, Long Island, or Westchester County, New York; Seattle, Washington; or Los Angeles, California; lack regular internet access; lack access to or ability to use a smartphone; not fluent in English; self-report cognitive impairment; unable or unwilling to provide informed consent.
8. Study Type (e.g., interventional, observational, etc.): Interventional
   - Allocation: Randomized
   - Intervention Model: Parallel assignment
   - Masking: No
   - Primary Purpose: Supportive Care
   - Type: Behavioral (e.g., Psychotherapy, Lifestyle Counseling)
   - Study Phase: Phase 1
9. Date of First Enrollment (anticipated or actual): September 24, 2025 (Actual)
10. Target Sample Size: N=80 family caregivers
11. Recruitment Status: Recruiting
12. Primary Outcome(s):
    - **Feasibility as measured by recruitment, retention, eligibility, and app usage metrics:**

Feasibility of the CarePair study will be assessed using multiple indicators across enrollment and follow-up: (1) Recruitment feasibility will be measured by the proportion of family caregivers who complete the screening survey among those invited, the proportion deemed eligible based on screening criteria, and the proportion who consent to participate and are enrolled, as well as the proportion of eligible caregivers who decline participation; (2) Retention feasibility will be evaluated by recording the number and proportion of participants who complete the study through the 6-week follow-up, including the number lost to follow-up and the number who withdraw after enrollment; and (3) Application feasibility will be assessed using backend analytics, including the number of app logins per participant and the number of interactions with CarePair features (e.g., reviewing, rating, saving, or notetaking), relative to the recommended minimum of 12 logins/interactions (2x/week over six weeks). In addition, application usability will be evaluated at follow-up using validated measures, including the System Usability Scale (SUS; scores range from 0-100, with higher scores indicating greater usability and a threshold of 68 indicating adequate usability) and the user version of the Mobile App Rating Scale (uMARS), which assesses engagement, functionality, aesthetics, and information quality (scores range from 20-100, with higher scores indicating more favorable evaluations).

- - - **Time Frame**: Enrollment – 6 Weeks
  - **Acceptability as measured by quantitative and qualitative survey feedback**: Acceptability of the CarePair application will be assessed at the 6-week follow-up using both quantitative and qualitative measures. Participants will complete a survey assessing satisfaction, engagement, perceived usefulness, and overall acceptability of the application (e.g., agreement with statements such as “I enjoyed my experience using the CarePair application”), with items rated on a 5-point Likert scale from 1=Strongly Disagree to 5=Strongly Agree and higher total scores indicating greater acceptability. In addition, participants in the intervention group will respond to five open-ended questions to provide qualitative feedback on their user experience, including perceived strengths, challenges, and suggestions for improvement (e.g., “Would you continue using the CarePair application after this study? Why or why not?”).
    - **Time Frame**: 6 Weeks

1. Key Secondary Outcome(s):
   - **Change in Caregiver Needs as measured by a 12-item Needs Assessment:** Family caregivers’ care-related needs (e.g., legal guidance, social support, education) will be assessed using a 12-item caregiver needs assessment, with total scores ranging from 12–48 and higher scores indicating greater caregiver needs.
     - **Time Frame**: Baseline, 6 Weeks
   - **Change in Caregiving Self-Efficacy as measured by the Revised Scale for Caregiving Self-Efficacy:** Caregiving self-efficacy will be measured using the 15-item Revised Scale for Caregiving Self-Efficacy, with total scores ranging from 0–100 and higher scores indicating greater perceived self-efficacy.
     - **Time Frame:** Baseline, 6 Weeks
   - **Change in Depressive Symptoms as measured by the Patient Health Questionnaire-9 (PHQ-9):** Caregiver depressive symptoms will be assessed using the PHQ-9, with total scores ranging from 0–27 and higher scores indicating greater severity of depressive symptoms.
     - **Time Frame:** Baseline, 6 Weeks
   - **Change in Anxiety as measured by the Generalized Anxiety Disorder-7 (GAD-7):** Caregiver anxiety will be measured using the GAD-7, with total scores ranging from 0–21 and higher scores indicating greater levels of anxiety.
     - **Time Frame:** Baseline, 6 Weeks
   - **Change in Caregiver Stress as measured by the Kingston Caregiver Stress Scale (KCSS):** Family caregiver stress will be assessed using the KCSS, with total scores ranging from 20–50 and higher scores indicating greater caregiver stress.
     - **Time Frame:** Baseline, 6 Weeks
   - **Change in Quality of Life as measured by the Satisfaction with Life Scale-5 (SWLS-5):** Caregiver quality of life will be assessed using the 5-item SWLS, with total scores ranging from 5–35, a score of 20 representing a neutral point, and higher scores indicating greater life satisfaction.
     - **Time Frame:** Baseline, 6 Weeks
2. Ethics Review:
   - Status: Approved
   - Date of primary approval: September 10, 2025
   - Date of most recent approval: September 10, 2025
   - Name and contact details of Ethics committee(s):

Name: University of Southern California Institutional Review Board

Address: 3720 S. Flower Street, Suite 325

Los Angeles, CA 90089

Telephone: 323-442-0114

1. Completion date: Not yet completed
2. Summary Results: Not applicable as the study has not been completed yet.
3. IPD Sharing Statement: Data sharing is not applicable to this article as no data were analyzed or generated. Upon completion of the project’s study aims, data will be available upon reasonable request.
