# Supplementary material for: A Mobile Self-Assessment and Referral Platform for Family Caregivers of Individuals With Alzheimer Disease and Related Dementias: Protocol for a Pilot Randomized Controlled Trial
Source: JMIR Res Protoc. 2026 Apr 1;15:e90244. doi: 10.2196/90244 (PMC13043018; doi:10.2196/90244)
Supplement: Multimedia Appendix 3 [file resprot-v15-e90244-s003.pdf]

University of Southern California
Leonard Davis School of Gerontology
3715 McClintock Ave. Los Angeles, CA 90089

Informed Consent for Research

Study Title: Testing & Refinement of CarePair: An Assessment and Referral Platform to Support Family Caregivers of Alzheimer's Disease and Related Dementias

Principal Investigator: Francesca Falzarano, PhD

Department: Leonard Davis School of Gerontology

Study ID: UP-25-00690 Valid From: 9/10/2025

Introduction

We invite you to take part in a research study focused on dementia family caregivers. Please take as much time as you need to read the consent form. You may want to discuss it with your family, friends, or your personal doctor. If you find any of the language difficult to understand, please ask questions. If you decide to participate, you will be asked to sign this form. A copy of the signed form will also be provided to you for your records.

Key Information

The following is a short summary of this study to help you decide whether you should participate. More detailed information is listed later in this form.

1) Being in this research study is voluntary—it is your choice.

2) You are being invited to participate in this study because you have been identified as a primary caregiver for a family member or friend with Alzheimer's disease or a related dementia (ADRD). The purpose of this study is to evaluate the feasibility, acceptability, and preliminary efficacy of the CarePair mobile application. CarePair is a self-assessment and referral platform to connect ADRD family caregivers with personalized resource recommendations tailored to their needs and preferences.

3) Participation in this study will last six weeks. During this time, you will be asked to log in and engage with ADRD- and/or caregiving-specific resources on your assigned online platform twice weekly. You will also be asked to complete two longer surveys—one at the beginning and one at the end of the study—and two brief check-in surveys during your participation. Intervention group participants may be invited to take part in an optional interview at the end of the study to share feedback on your experiences with the *CarePair* app. All study procedures will be conducted virtually.

4) There are risks from participating in this study. The most common risks are possible emotional discomfort from surveys, interviews, and/or ADRD-/caregiving-related content,

minor frustration while using the online platform, and a potential, yet minimal, risk of breach of confidentiality. More detailed information about the risks of this study can be found under the “Risk and Discomfort” section.

5) You may not receive any direct benefit from taking part in this study. However, your participation in this study may help us learn ways to improve and develop effective digital tools that connect dementia family caregivers with needed services and supports.

6) If you decide not to participate in this research, your other choice may include not participating.

Purpose

This study aims to evaluate the feasibility, acceptability, and preliminary effects of **CarePair**, a mobile application designed to support family caregivers of individuals living with dementia. CarePair is a self-assessment and referral platform that aims to connect caregivers with personalized resources aligned with their preferences and caregiving needs. You are invited as a possible participant because you have been identified as the primary caregiver of a family member or friend living with Alzheimer’s disease or a related dementia (ADRD). About 80 participants will take part in the study. This research is being funded by the National Institute on Aging (NIA).

Procedures

If you decide to participate, this is what will happen:

- Before today’s meeting, you should have already completed a brief screening survey to determine your eligibility. Now, we are reviewing the consent form together and answering any questions you may have. If you agree to participate after this meeting, you will begin by completing an online baseline survey, which should take about 30–45 minutes to complete.
- After completing the baseline survey, you will be randomly assigned to one of two study groups. This randomization process is like flipping a coin—neither you nor the research team can choose which group you are placed in. You will have a one out of two chance of being placed in either group.
    - If you are assigned to the first group, you will receive training materials and access to the CarePair mobile application, which includes a library of articles and services designed to support your unique needs. You will be asked to use the app at least two times per week for six weeks.

  - If you are assigned to the second group, you will receive access to a digital folder with ADRD/caregiver-focused articles. You will be asked to engage with this material at least two times per week for six weeks.
- During your six weeks of participation in the study, you will receive weekly emails from the study team. These will alternate between check-in messages and brief 5–10-minute check-in surveys for you to complete.
- At the end of the six-week period, you will also be asked to complete a follow-up survey, which should take approximately 30–45 minutes to complete.
- If you are randomized to the intervention group, you may also be invited to take part in an optional 15–30-minute interview to share your feedback on your experience using the *CarePair* app.

Risk and Discomforts

Possible risks and discomforts you could experience during this study include:

Use of the CarePair Mobile Application *(Group One Only)*
Using the CarePair app may result in minimal discomfort, such as frustration if the app does not work as expected or if navigating the features feels confusing or time-consuming. You will be asked to read and engage with articles within the app, and are encouraged, but not required, to explore the suggested services based on your interests and needs. Some app content may relate to emotionally sensitive topics such as grief or guilt related to caregiving, which could evoke distress. You may choose not to engage with any articles or services that make you feel uncomfortable or distressed.

Study ID: UP-25-00690 Valid From: 9/10/2025

You are free to stop using the app at any time during the study. We just ask that you notify a member of the study team if you decide to discontinue use, as this will also end your study participation.

Digital Attention Control Materials *(Group Two Only)*
Participants in the second group will be asked to read educational articles related to ADRD/caregiving. Some content may address emotionally sensitive topics such as caregiving, dementia, or end-of-life care, which could cause mild distress. You are free to skip any material that feels uncomfortable, as you will be able to choose which content you engage with.

These materials will be provided as PDF files in a secure, password-protected online folder. You will access the folder using a unique study ID, not your name nor any personal information, so your identity will not be linked to the platform. Information linking your study ID to your name will be stored separately in a password-protected REDCap database with two-factor authentication (2FA), accessible only to authorized study personnel.

Surveys
Some of the survey questions may make you feel uncomfortable or bring up difficult emotions related to caregiving or dementia. You may choose to skip any questions you do not wish to answer.

Optional Interview *(Group One Only)*
If you are invited to take part in an optional feedback interview, you will be asked to reflect on your experience using the CarePair app. This could bring up emotional topics or personal reflections that could bring about discomfort. You can choose to skip any question or stop the interview at any time.

Privacy and Confidentiality
As with any research study, there is a potential risk of a breach of confidentiality. However, we believe this risk is minimal. We will take several precautions to protect your personal information, including secure storage of all study data and the use of unique ID codes in place of names. The link between your identity and your study ID will be stored separately in a password-protected REDCap database with 2FA, accessible only to authorized study personnel. No identifying information will be included in any reports, publications, or presentations resulting from this study.

Surveys/Questionnaires/Interviews

Some of the questions may make you feel uneasy or embarrassed. You can choose to skip or stop answering any questions you don’t want to.

Breach of Confidentiality

There is a small risk that people not connected with this study will learn your identity or personal information.

Study ID: UP-25-00690 Valid From: 9/10/2025

Unforeseen Risks

There may be other risks that are not known at this time.

Risk of sharing data

We will do our best to protect your data during storage and when they are shared. However, there remains a possibility that someone could identify you. There is also the possibility that unauthorized people might access your data. In either case, we cannot reduce the risk to zero.

Benefits

There are no direct benefits to you from taking part in this study. However, your involvement may help researchers improve and develop effective tools that connect dementia family caregivers with needed services and supports—ultimately aiming to enhance caregiver well-being and quality of life.

Privacy/Confidentiality

We will keep your records for this study confidential as far as permitted by law. However, if we are required to do so by law, we will disclose confidential information about you. Efforts will be made to limit the use and disclosure of your personal information, including research study and medical records, to people who are required to review this information. We may publish the information learned from this study in scientific journals or present it at meetings. If we do, we will not use your name or any other identifying information.

The University of Southern California's Institutional Review Board (IRB) and Human Subjects' Protections Program (HSPP) may review your records. Organizations that may also inspect and copy your information include:

- The University of Southern California (USC)
- The USC Institutional Review Board (IRB)
- The Office of Human Research Protection (OHRP)
- Department of Health and Human Services
- National Institutes of Health (NIH)
- The Food and Drug Administration (FDA) and/or their representatives

Study ID: UP-25-00690 Valid From: 9/10/2025

- The NIH/NIA and/or their representative will have access to your files.

Future use of data

Your data will be maintained confidentially and may be shared with other researchers. The research may be about similar or unrelated topics to this study. Our goal is to make more research possible. We plan to keep your data indefinitely. If shared, data will be transferred securely. If you are not comfortable with this, you should not participate in this study.

This research is covered by a Certificate of Confidentiality from the National Institutes of Health. This means that the researchers cannot release or use information, documents, or samples that may identify you in any action or suit unless you say it is okay. They also cannot provide them as evidence unless you have agreed. This protection includes federal, state, or local civil, criminal, administrative, legislative, or other proceedings. An example would be a court subpoena.

There are some important things that you need to know. The Certificate DOES NOT stop reporting that federal, state, or local laws require. Some examples are laws that require reporting of child or elder abuse, some communicable diseases, and threats to harm yourself or others. The Certificate CANNOT BE USED to stop a sponsoring United States federal or state government agency from checking records or evaluating programs. The Certificate DOES NOT stop disclosures required by the federal Food and Drug Administration (FDA). The Certificate also DOES NOT prevent your information from being used for other research if allowed by federal regulations. Researchers may release information about you when you say it is okay. For example, you may give them permission to release information to insurers, medical providers or any other persons not connected with the research. The Certificate of Confidentiality does not stop you from willingly releasing information about your involvement in this research. It also does not prevent you from having access to your own information.

This study will use ResearchMatch.org for recruitment purposes. To understand the privacy and confidentiality limitations associated with using ResearchMatch we strongly advise you to familiarize yourself with ResearchMatch's privacy policy. USC has no jurisdiction or oversight of how data are used or shared on third party applications.

Alternatives

An alternative would be to not participate in this study.

Payments/Compensation

Individuals who complete the baseline and follow-up surveys will receive a $25 Amazon.com digital gift card (or equivalent payment method) after completing each assessment, for a total compensation of $50 for completing all required study activities. Participants who are invited to and choose to complete the optional follow-up interview will receive an additional $20 Amazon gift card. All payments will be processed and

Study ID: UP-25-00690 Valid From: 9/10/2025

emailed within 1–2 business days, and typically no later than one week after completing the corresponding study activity.

Payments for research participation are considered taxable income and participants may be required to pay taxes on this income. If participants are paid $600 or more in total within a calendar year for participation in one or more research studies, the University will report this as income to the IRS and participants may receive an Internal Revenue Service (IRS) Form 1099. This does not include any payments you receive to pay you back for expenses like parking fees.

Cost

The study will pay for all research activities. You and/or your health plan/insurance will not be billed for any research activities.

New Information

We will tell you about any new information that may affect your health, welfare, or willingness to stay in the research.

Voluntary Participation

It is your choice whether to participate. If you choose to participate, you may change your mind and leave the study anytime. If you decide not to participate, or choose to end your participation in this study, you will not be penalized or lose any benefits you are otherwise entitled to.

Withdrawal from Study Instructions

You can stop participating at any time. However, if you decide to stop participating in the study, we encourage you to notify and talk to a study team member first.

Participant Termination

You may be removed from this study without your consent for any of the following reasons: you do not follow the study investigator's instructions (e.g., completing required study activities; responding to study personnel within 3 weeks), at the discretion of the study investigator or the sponsor, or the sponsor closes the study. If this happens, the study investigator will inform you.

Contact Information

If you have questions, concerns, complaints, or think the research has hurt you, talk to the Principal Investigator, Francesca Falzarano, PhD at falzaran@usc.edu, or 213-740-0357.

Study ID: UP-25-00690 Valid From: 9/10/2025

This research has been reviewed by the USC Institutional Review Board (IRB). The IRB is a research review board that reviews and monitors research studies to protect the rights and welfare of research participants. Contact the IRB if you have questions about your rights as a research participant or you have complaints about the research. You may contact the IRB at (323) 442-0114 or by email at hrpp@usc.edu.

Statement of Consent

I have read (or someone has read to me) the information provided above. I have been given a chance to ask questions. All my questions have been answered. By signing this form, I am agreeing to take part in this study.

Name of Research Participant	Signature	Date Signed (and Time*)

Person Obtaining Consent

I have personally explained the research to the participant using non-technical language. I have answered all the participant's questions. I believe that the participant understands the information described in this informed consent and freely consents to participate.

Name of Person Obtaining Informed Consent	Signature	Date Signed (and Time*)

Study ID: UP-25-00690 Valid From: 9/10/2025
