# Supplementary material for: A Mobile Self-Assessment and Referral Platform for Family Caregivers of Individuals With Alzheimer Disease and Related Dementias: Protocol for a Pilot Randomized Controlled Trial
Source: JMIR Res Protoc. 2026 Apr 1;15:e90244. doi: 10.2196/90244 (PMC13043018; doi:10.2196/90244)
Supplement: Peer Review Report 1 [file resprot-v15-e90244-s005.pdf]

SUMMARY STATEMENT
(Privileged Communication)

PROGRAM CONTACT:
Melissa Gerald
301-402-4156
geraldmel@nia.nih.gov

Release Date: 03/05/2021
Revised Date:

Application Number: 1 K99 AG073509-01

Principal Investigator

FALZARANO, FRANCESCA

Applicant Organization: WEILL MEDICAL COLL OF CORNELL UNIV

Review Group: NIA-S
Behavior and Social Science of Aging Review Committee
NIA-S

Meeting Date: 02/04/2021
Council: MAY 2021
Requested Start: 07/01/2021
RFA/PA: PA20-187
PCC: 2BFAMGE

Project Title: The Caregiver Resource Room: Enhancing Support for Caregivers of Alzheimer's Disease and Related Dementias (ADRD)
SRG Action: Impact Score:31
Next Steps: Visit https://grants.nih.gov/grants/next_steps.htm
Human Subjects: 30-Human subjects involved - Certified, no SRG concerns
Animal Subjects: 10-No live vertebrate animals involved for competing appl.
Gender: 1A-Both genders, scientifically acceptable
Minority: 1A-Minorities and non-minorities, scientifically acceptable
Age: 3A-No children included, scientifically acceptable

Project Year	Direct Costs Requested	Estimated Total Cost
1	110,236	112,264
2	112,793	114,868
3	249,000	253,580
4	249,000	253,580
5	249,000	253,580
TOTAL	970,029	987,871

ADMINISTRATIVE BUDGET NOTE: The budget shown is the requested budget and has not been adjusted to reflect any recommendations made by reviewers. If an award is planned, the costs will be calculated by Institute grants management staff based on the recommendations outlined below in the COMMITTEE BUDGET RECOMMENDATIONS section.

RESUME AND SUMMARY OF DISCUSSION: This application, for a Pathway to Independence Award (K99/R00), is submitted by Weill Medical College of Cornell University (WMC-CU), on behalf of the candidate, Dr. Francesca Falzarano. It requests five years of support to develop and evaluate tools that are informed by empirical evidence, designed to improve outcomes in ADRD caregivers, and empower caregivers with the resources to address their psychosocial needs and to effectively provide care to persons with dementia (PwD). The candidate is a NIA T32 Post-Doctoral Research Fellow in the Division of Geriatrics and Palliative Medicine at WMC, collaborating with Drs. Holly Prigerson, Sara Czaja and Karl Pillemer as co-mentors. The candidate received a Ph.D. (2019) in Applied Developmental Psychology at Fordham University. The application builds upon the candidate's research and will develop additional skillsets that are integral to Dr. Falzarano's career development to independence: develop and test behavioral interventions rooted in technology; address barriers to accessing supportive resources in dementia caregivers; and translate research findings into tangible strategies to increase service use in ADRD caregivers. Notably, findings from this K-award have the potential to serve as the foundation for future R01 grant application submissions during the R00 phase. The candidate has 11 publications (5 first author), a Donahue Foundation grant award, and scientific presentations. She has assembled a strong team of Co-Mentors with a supportive academic institution. Concerns encompass an overly ambitious training plan given the short two-year timeline of the K99 phase. The candidate describes the tool development and evaluation process used to test the acceptability and feasibility of the intervention with caregivers. In the application it is unclear why machine learning was a necessary aspect of the application given the small sample size. The final result of R00 phase is essentially a website. How will the candidate assure that knowledge of the website is widely disseminated lacks clarity. The strategy or details on how the website will be updated or monitored is absent from the application. The research plan may be overly ambitious in the inclusion of machine learning algorithms and technology development. Additional justification is required for the use of machine learning (ML) for the proposed sample size (20 structured interviews). These weaknesses temper enthusiasm for this application.

TRAINING IN THE RESPONSIBLE CONDUCT OF RESEARCH: Acceptable. The planned activities satisfy the requirement for training in the responsible conduct of research.

DESCRIPTION (provided by candidate): With nearly 6 million cases of Alzheimer's disease and related dementias (ADRD) diagnosed in the United States in 2019, the health and well-being of ADRD caregivers is of critical importance. Promoting the health and well-being of ADRD caregivers is an effective way to support these unpaid staples of ADRD care. Although interventions targeting ADRD caregivers continues to accelerate, little attention has been paid to caregivers' use of supportive services, which remains strikingly low. The objective of this K99/R00 application is to enable Dr. Falzarano to develop the necessary expertise to design, implement, and evaluate studies focused on leveraging technology to support and address ADRD caregivers' multifarious needs. In the K99 phase, Dr. Falzarano will work toward achieving five training objectives that will prepare her to transition to become an independent investigator who designs and evaluates evidence-based technologies to enhance tailored caregiver support. First, she will extend her knowledge in substantive areas including dementia caregiving and front-end and back-end software development. Second, she will learn how to develop, implement, and test behavioral interventions with an emphasis on collaboration with community partners. Third, she will receive training in statistical methods to examine feasibility, acceptability, and preliminary effectiveness in randomized controlled trials (RCTs). Fourth, she will learn about measurement development and psychometric validation of assessments for ADRD caregivers. Fifth, Dr. Falzarano will pursue professional development activities, particularly in the areas of grant writing, leadership, and integration of research and web development teams. The four proposed research aims were chosen to complement the areas identified for career development. Aim 1a will provide the requisite training and experiences to provide Dr. Falzarano with the critical skills needed to conduct the proposed study. Aim 1b will include interviews with ADRD caregivers to identify needs, awareness of, barriers to, and willingness to use services. Aim 1c will leverage key stakeholder

(dementia-care experts) input to identify existing services corresponding to areas of need identified in Aim 1b. Aim 2 will leverage data collected from caregivers and stakeholders to inform the iterative development and design of the Caregiver Resource Room (CRR) website, which will include a digital self-assessment tool using machine learning models to identify and categorize needs and provide an output of targeted service recommendations. Aim 3 will include an analysis of the tool's content, design, features, and services output, which will be iteratively refined based on ADRD caregiver feedback. A randomized controlled trial will be conducted in Aim 4 to determine the feasibility, acceptability, and preliminary efficacy of the CRR as a tool to enhance awareness of and access to relevant services to address unmet needs and improve mental health. The proposed research is well-aligned with the NIA's strategic initiative to foster the development of research scientists in aging and to develop and test promising interventions to improve ADRD caregiver outcomes.

PUBLIC HEALTH RELEVANCE: The multifarious needs of ADRD caregivers often remain unaddressed, and the utilization rate of supportive services remains strikingly low, with many caregivers uncertain about what services may be right for them, what exists, and how to access them. There is a need for a robust, reliable tool that comprehensively evaluates ADRD caregiver needs with a mechanism to connect needs with existing services, thereby overcoming barriers that ADRD caregivers face in accessing needed supportive care. The proposed project seeks to iteratively develop and test an online self-assessment and tailored referral platform, grounded in machine learning principles and informed by stakeholder perspectives, to address needs and enhance knowledge and use of relevant services, with the overall goal of linking ADRD caregivers with relevant support and thereby promoting their mental health and well-being.

DISCLAIMER: Please note that the following critiques were prepared by the reviewers prior to the Study Section meeting and are provided in an essentially unedited form. While there is opportunity for the reviewers to update or revise their written evaluation, based upon the group's discussion, there is no guarantee that individual critiques have been updated subsequent to the discussion at the meeting. Therefore, the critiques may not fully reflect the final opinions of the individual reviewers at the close of group discussion or the final majority opinion of the group. Thus, the Resume and Summary of Discussion is the final word on what the reviewers actually considered critical at the meeting.

CRITIQUE 1:

Candidate: 1
Career Development Plan/Career Goals/Plan to Provide Mentoring: 2
Research Plan: 2
Mentor(s), Co-Mentor(s), Consultant(s), Collaborator(s): 1
Environment, Commitment to the Candidate: 1

Overall Impact:
The candidate, Dr. Francesca Falzarano, is an applied developmental psychologist with experience in caregiving. The candidate's work in this space began after serving as a caregiver. The candidate has successful track record as a junior investigator and has a well-established mentoring team. The career development plan that takes into consideration expertise of mentors and areas of growth needed beyond their skills for Dr. Falzarano to complete the activities designed in this application. Phase 1 is the K99 training plan provides insights into areas of growth on machine learning and caregiving. The skill acquisition mentioned will foster the development of caregiving interventions. Phase 2 is the intervention development. The candidate describes the tool development and evaluation process used to test the acceptability and feasibility of the intervention with caregivers. In the application it was unclear on why machine learning was necessary aspect of the application given the small sample size. There is also an opportunity to connect directly with a technology developer through this research to

build on team-based science rather than Dr. Falzarano focusing on technology development part of the application. This will build on the skills of translational science.

1. Candidate:
Strengths
- Dr. Falzarano is an applied developmental psychologist with experience in caregiving (personal and professionally).
- Over the past year, the candidate has successfully authored 11 papers in the area of interest and this research will expand on that knowledge base.
- The candidate has experience using theoretical foundations as well as mixed methods research.
- The candidate has experience in cognitive assessment techniques and community collaborations.

Weaknesses
- None noted.

2. Career Development Plan/Career Goals & Objectives:
Strengths
- Well-developed career development plan that takes into consideration expertise of mentors.
- Details on programs external to the candidate's home institution to offset gaps in the mentors' expertise.
- The five training goals partner well with the current level of experience.

Weaknesses
- A concern in the training in the training is the nuance given to caregiving and technology training. While this connection is important it seems that the training objective for 1 should relate directly to expanding existing skills in caregiving research to technology.

3. Research Plan:
Strengths
- Dr. Falzarano research plan builds on the foundation of completed work examining caregiver utilization of support resources.
- From Dr. Falzarano's previous work the candidate learned that less than 10% of caregivers used support resources to assist with caregiving for PwD.
- The plan has 2 overall phases: Phase 1 is the K99 training plan, the plan is well detailed and provides insights areas of growth that Dr. Falzarano needs to expand on their research with machine learning and caregiving. The skill acquisition for developing caregiving interventions is appropriate and fits with the next steps to advance their career. Phase 2 is the intervention development. The process described builds on each other. Excellent description of tool development, conducting task analysis, and conducting the acceptability and feasibility study of the intervention.
- The connection that is made during the first part of the K99 leading to the R00 allow for Dr. Falzarano to gain insight on barriers and facilitators to the use of a caregiver intervention to assist with stress reduction, burnout, and self-care while addressing the needs of the PwD.

Weaknesses
- Not provided.

4. Mentor(s), Co-Mentor(s), Consultant(s), Collaborator(s):
Strengths
- Primary mentor, Dr. Holly Prigerson, is well-suited to assist Dr. Francesca Falzarano in the next steps in their career. Dr. Prigerson is an established investigator that has experience with caregiving quality of life and end-of-life care.
- The secondary mentor, Dr. Sara Czaja, has expertise in aging and technology.

Weaknesses
- None noted.

5. Environment and Institutional Commitment to the Candidate:
Strengths
- Appropriate and supportive of this research:

Weaknesses
- None noted.

Study Timeline:
Strengths
- Appropriate as outlined.

Weaknesses
- None noted.

Protections for Human Subjects:
Acceptable Risks and Adequate Protections.

Data and Safety Monitoring Plan:
Acceptable.

Inclusion Plans:
- Sex/Gender: Distribution justified scientifically.
- Race/Ethnicity: Distribution justified scientifically.
- Inclusion/Exclusion of Children under 18: Excluding ages <18; justified scientifically.

Training in the Responsible Conduct of Research:
Acceptable.
Comments on Format:
- acceptable.

Comments on Subject Matter:
- Acceptable.

Comments on Faculty Participation:
- Acceptable.

Comments on Duration:
- Acceptable.

Comments on Frequency:
- Acceptable.

Resource Sharing Plans:
Acceptable.

Budget and Period of Support:
Recommend as Requested.

CRITIQUE 2:

Candidate: 2
Career Development Plan/Career Goals/Plan to Provide Mentoring: 3
Research Plan: 4
Mentor(s), Co-Mentor(s), Consultant(s), Collaborator(s): 2

Environment, Commitment to the Candidate: 2

Overall Impact:
The candidate, Dr. Francesca Falzarano, is in the behavioral geriatrics program at Weill Cornell Medical College with a Ph.D. in psychology from Fordham University. Research is motivated in part by her own experience caregiving for mother with Cortical Basal Ganglionic Degeneration. Candidate has shown passion for the area of ADRD caregiving and has achieved appropriate training and expertise in the research area for this stage of career. Scientific output shows track record of productivity and ability to form community partnerships. It appears that the candidate strongly desires to incorporate machine lacking. Not sure how machine learning fits in given the small and straightforward data source from structured interviews seems simple regression models could suffice. Again, it appears candidate had an a priori interest in machine learning, but the proposed research plan does not require it. Overall, candidate appears promising, and training and research plans are appropriate for her level and achievable. More justification is necessary for some of the components.

1. Candidate:
Strengths
- Highly productive, in first year of post-doctoral, 11 publications (5 first author), with 4 manuscripts in preparation, and a foundation grant awarded by the Donahue Foundation.
- Good deal of research experience and engagement of community partners.
- Personal experience as a caregiver clear passion for the topic.

Weaknesses
- Not provided.

2. Career Development Plan/Career Goals & Objectives/Plan to Provide Mentoring:
Strengths
- Activities for each training goal clearly laid out.
- Timeline for transition to independent phase is appropriate.
- If goals of plan are achieved, candidate would possess quite a tool belt of research and tech development skills.

Weaknesses
- 5 training objectives, some of which are rather broad; could be more focused for a two-year K99.
- Because justification is needed for why machine learning is necessary for this research plan, it raises the question of whether ML training is necessary.
- Stronger case needs to be made as to why candidate requires training in ML (even if it is necessary for research plan) and software development, when it may be more efficient to collaborate/contract with persons trained in those areas to achieve research goals.
- Following up on previous point, candidate may be seeking overly broad training in too many areas, rather than focusing on the aspect of this research aim that she can contribute the most towards. The overall goal is comprehensive needs assessment for CGs and linking them to available services, web technology may be useful for this purpose, but questionable whether candidate has to develop that software herself.

3. Research Plan:
Strengths
- Each step in the research plan and justification for it is very clearly laid out.
- Addressing ADRD caregivers' unmet needs and linking them to relevant support is a significant goal.

Weaknesses
- Four overall aims, with 3 Sub-Aims for Aim 1 in the K99 portion, may be overly ambitious.

- How does this improve on existing web services like Alzheimer's disease you need lacks clarity? What is innovative about this particular service lacks clarity.

4. Mentor(s), Co-Mentor(s), Consultant(s), Collaborator(s):
Strengths
- Primary mentor won the Harvard University Medical School (HMS) A. Clifford Barger Award for Excellence in Mentoring and was a founding member of the HMS Council of Mentors; has mentored over 90 junior investigators, including over 25 successful K-applications.
- Mentorship team has strong track record of training independent researchers and expertise in the areas of caregiving and technology needed for this project.

Weaknesses
- Expertise of mentorship team is similar and overlaps.

5. Environment and Institutional Commitment to the Candidate:
Strengths
- Strong environment for ADRD caregiving research. Commitment from institution and department.
- Commitment from Caring Kind and Alz You Need for CAB and caregiver recruitment.

Weaknesses
- Not provided.

Protections for Human Subjects:
Acceptable Risks and Adequate Protections.
- Adequate for interview and focus-based research.

Inclusion Plans:
- Sex/Gender: Distribution justified scientifically.
- Race/Ethnicity: Distribution justified scientifically.
- Inclusion/Exclusion of Children under 18: Excluding ages <18; justified scientifically.
- Adult caregivers is focus.

Training in the Responsible Conduct of Research:
Acceptable.
Comments on Format:
- RCR course & faculty mentorship.

Comments on Subject Matter:
- Appropriate.

Comments on Faculty Participation:
- Full commitment from primary mentor.

Comments on Duration:
- Appropriate.

Comments on Frequency:
- Appropriate.

Resource Sharing Plans:
Acceptable.
- Highly detailed plan.

Budget and Period of Support:
Recommend as Requested.

CRITIQUE 3:

Candidate: 2
Career Development Plan/Career Goals/Plan to Provide Mentoring: 3
Research Plan: 4
Mentor(s), Co-Mentor(s), Consultant(s), Collaborator(s): 1
Environment, Commitment to the Candidate: 1

Overall Impact:
While the proposed research will provide appropriate training for the candidate, Dr. Francesca Falzarano, the research is not innovative given the team of stellar mentors and the institutional resources available.

1. Candidate:
Strengths
- Strong interest in the research area, aging and caregiving with 11 publications.
- Well published.
- Productive and hard working.

Weaknesses
- 11 publications in 1 year lacks clarity.

2. Career Development Plan/Career Goals & Objectives/Plan to Provide Mentoring:
Strengths
- Mentors.
- Logical objectives.

Weaknesses
- There are too many scheduled meetings with many mentors (Table 1).

3. Research Plan:
Strengths
- The Caregiver Resource Room sounds useful.

Weaknesses
- Justification for sample size.
- Lack of attention to services and how to measure them.
- Final product as described isn’t impressive.
- The website versus usual care appear weak for RCT.
- Model outcomes lacks clarity.

4. Mentor(s), Co-Mentor(s), Consultant(s), Collaborator(s):
Strengths
- Superb.
- Good collaborators.

Weaknesses
- None noted.

5. Environment and Institutional Commitment to the Candidate:
Strengths
- Superb environment.
- Current postdoc fits the mechanism.

Weaknesses
- Will the candidate receive a faculty position if not for this application lacks clarity?

Budget and Period of Support:
Recommended budget modifications or possible overlap identified:

- First two years are strong; the last three years are weak.

THE FOLLOWING SECTIONS WERE PREPARED BY THE SCIENTIFIC REVIEW OFFICER TO SUMMARIZE THE OUTCOME OF DISCUSSIONS OF THE REVIEW COMMITTEE, OR REVIEWERS' WRITTEN CRITIQUES, ON THE FOLLOWING ISSUES:

PROTECTION OF HUMAN SUBJECTS: ACCEPTABLE. Acceptable Risks and/or Adequate Protections.

INCLUSION OF WOMEN PLAN: ACCEPTABLE. The team will not exclude participants based on gender. Published estimates indicate that approximately 61% of family caregivers are women. Based on these published data and data reflecting recruitment sites' family caregiver distribution, it is expected that of the family caregivers enrolled in the proposed research, approximately 60-70% of the sample will be female and 30-40% will be male. Additionally, based on data from participating sites, approximately 50% of the key stakeholder sample is expected to consist of females.

INCLUSION OF MINORITIES PLAN: ACCEPTABLE. Family caregivers will also be purposefully selected to reflect participating sites', including Weill Cornell Medicine's Center on Aging and CaringKind NYC, family caregiver distribution, ensuring adequate family caregiver racial and ethnic representation. Based on these data, it is estimated that approximately 60% of the sample will be Caucasian, 30% will be Black or African American, 30% Latinx/Hispanic, and 10% Asian. To address concerns regarding underrepresentation of minorities, the team will make every effort to prioritize the recruitment of racial and ethnic minorities into the study.

INCLUSION ACROSS THE LIFESPAN: ACCEPTABLE. This study will recruit primary dementia caregivers of community dwelling of persons with dementia (PwD) aged 18-99 years of age.

COMMITTEE BUDGET RECOMMENDATIONS: The budget was recommended as requested.

Footnotes for 1 K99 AG073509-01; PI Name: Falzarano, Francesca

NIH has modified its policy regarding the receipt of resubmissions (amended applications).See Guide Notice NOT-OD-18-197 at https://grants.nih.gov/grants/guide/notice-files/NOT-OD-18-197.html. The impact/priority score is calculated after discussion of an application by averaging the overall scores (1-9) given by all voting reviewers on the committee and multiplying by 10. The criterion scores are submitted prior to the meeting by the individual reviewers assigned to an application, and are not discussed specifically at the review meeting or calculated into the overall impact score. Some applications also receive a percentile ranking. For details on the review process, see http://grants.nih.gov/grants/peer_review_process.htm#scoring.

MEETING ROSTER

Behavior and Social Science of Aging Review Committee
National Institute on Aging Initial Review Group
NATIONAL INSTITUTE ON AGING
NIA-S

NIA-S
02/04/2021 - 02/05/2021

Notice of NIH Policy to All Applicants: Meeting rosters are provided for information purposes only. Applicant investigators and institutional officials must not communicate directly with study section members about an application before or after the review. Failure to observe this policy will create a serious breach of integrity in the peer review process, and may lead to actions outlined in NOT-OD-14-073 at https://grants.nih.gov/grants/guide/notice-files/NOT-OD-14-073.html and NOT-OD-15-106 at https://grants.nih.gov/grants/guide/notice-files/NOT-OD-15-106.html, including removal of the application from immediate review.

CHAIRPERSON(S)

FITZPATRICK, ANNETTE L., PHD
RESEARCH PROFESSOR
DEPARTMENTS OF FAMILY MEDICINE,
EPIDEMIOLOGY, AND GLOBAL HEALTH
UNIVERSITY OF WASHINGTON
SEATTLE, WA 98115

MEMBERS

BALLS-BERRY, JOYCE E., PHD
ASSOCIATE PROFESSOR OF NEUROLOGY
ALZHEIMER'S DISEASE RESEARCH CENTER
WASHINGTON UNIVERSITY
ST. LOUIS, MO 63108

BARRY, LISA C., PHD, MPH
ASSOCIATE PROFESSOR
CENTER ON AGING AND DEPARTMENT OF PSYCHIATRY
UNIVERSITY OF CONNECTICUT HEALTH CENTER
FARMINGTON, CT 06030

BELL, RONNY A., PHD *
PROFESSOR
DEPARTMENT OF SOCIAL SCIENCES AND HEALTH POLICY
WAKE FOREST UNIVERSITY SCHOOL OF MEDICINE
WINSTON-SALEM, NC 27101

CHAPMAN, BENJAMIN P., PHD, MPH *
ASSOCIATE PROFESSOR
DEPARTMENTS OF PSYCHIATRY
AND PUBLIC HEALTH SCIENCES
UNIVERSITY OF ROCHESTER MEDICAL CENTER
ROCHESTER, NY 14642

DONG, XINQI, MD
PROFESSOR
INSTITUTE FOR HEALTH, HEALTH CARE POLICY
AND AGING RESEARCH
RUTGERS UNIVERSITY
NEW BRUNSWICK, NJ 08901

ELK, RONIT, PHD *
PROFESSOR
DIVISION OF GERIATRICS, GERONTOLOGY,
AND PALLIATIVE CARE
THE UNIVERSITY OF ALABAMA AT BIRMINGHAM
BIRMINGHAM, AL 35205

ENDERS, FELICITY T., PHD *
PROFESSOR AND HEAD
SECTION OF CLINICAL STATISTICS
MAYO CLINIC COLLEGE OF MEDICINE & SCIENCE
ROCHESTER, MN 55905

FAIN, MINDY J., MD *
PROFESSOR OF MEDICINE
CHIEF, DIVISION OF GERIATRICS, GENERAL INTERNAL
MEDICINE, AND PALLIATIVE MEDICINE
UNIVERSITY OF ARIZONA COLLEGE OF MEDICINE
TUCSON, AZ 85719

FLATT, JASON DANE, PHD, MPH *
ASSISTANT PROFESSOR
INSTITUTE FOR HEALTH AND AGING
UNIVERSITY OF CALIFORNIA, SAN FRANCISCO
SAN FRANCISCO, CA 94118

HEYN, PATRICIA C., PHD *
ASSOCIATE PROFESSOR
THE ROCKY MOUNTAIN ALZHEIMER'S DISEASE CENTER
UNIVERSITY OF COLORADO DENVER
DENVER, CO 80203

HILL, ANGELA M., PHMD *
PROFESSOR AND ASSOCIATE DEAN OF CLINICAL AFFAIRS
DEPARTMENT OF PHARMACY PRACTICE
TANEJA COLLEGE OF PHARMACY
UNIVERSITY OF SOUTH FLORIDA
TAMPA, FL 33620

JAMES, BRYAN DAVID, PHD *
ASSOCIATE PROFESSOR
ALZHEIMER'S DISEASE CENTER
RUSH UNIVERSITY
CHICAGO, IL 60612

JEAN-PIERRE, PASCAL, PHD, MPH *
ASSOCIATE PROFESSOR
BEHAVIORAL SCIENCES AND SOCIAL MEDICINE
FLORIDA STATE UNIVERSITY COLLEGE OF MEDICINE
TALLAHASSEE, FL 32306

JONES, VANYA C., PHD, MPH *
ASSOCIATE PROFESSOR
THE DEPARTMENT OF HEALTH, BEHAVIOR AND SOCIETY
JOHNS HOPKINS UNIVERSITY BLOOMBERG SCHOOL
OF PUBLIC HEALTH
BALTIMORE, MD 21205

MEARA, ELLEN R., PHD
PROFESSOR OF HEALTH ECONOMCIS AND POLICY
DEPARTMENT OF HEALTH POLICY AND MANAGEMENT
HARVARD UNIVERSITY T.H. CHAN SCHOOL
OF PUBLIC HEALTH
BOSTON, MA 02115

MISHRA, MANOJ K., PHD
PROFESSOR
DEPARTMENT OF BIOLOGICAL SCIENCES
ALABAMA STATE UNIVERSITY
MONTGOMERY, AL 36101

MONIN, JOAN E., PHD
ASSOCIATE PROFESSOR
SOCIAL AND BEHAVIORAL SCIENCES DEPARTMENT
YALE SCHOOL OF PUBLIC HEALTH
NEW HAVEN, CT 06520

SEGERSTROM, SUZANNE C., PHD, MPH
PROFESSOR
DEPARTMENT OF PSYCHOLOGY
UNIVERSITY RESEARCH
UNIVERSITY OF KENTUCKY
LEXINGTON, KY 40506

SIEGLER, ILENE C., PHD, MPH *
PROFESSOR
DEPARTMENT OF PSYCHIATRY
AND BEHAVIORAL SCIENCES
DUKE UNIVERSITY SCHOOL OF MEDICINE
DURHAM, NC 27710

SKIPPER, ANTONIUS D., PHD *
ASSISTANT PROFESSOR
THE GERONTOLOGY INSTITUTE
GEORGIA STATE UNIVERSITY
ATLANTA, GA 30303

TANG, YI-YUAN, PHD *
PROFESSOR AND CHAIR
DEPARTMENT OF PSYCHOLOGICAL SCIENCES
TEXAS TECH UNIVERSITY
LUBBOCK, TX 79409

TARRAF, WASSIM, PHD
ASSOCIATE PROFESSOR
INSTITUTE OF GERONTOLOGY
DEPARTMENT OF HEALTHCARE SCIENCES
WAYNE STATE UNIVERSITY
DETROIT, MI 48073

THORPE, ROLAND J. JR, PHD
PROFESSOR
DEPARTMENT OF HEALTH, BEHAVIOR, AND SOCIETY
JOHNS HOPKINS UNIVERSITY BLOOMBERG SCHOOL
OF PUBLIC HEALTH
BALTIMORE, MD 21205

ZELINSKI, ELIZABETH M., PHD *
PROFESSOR
RITA AND EDWARD POLUSKY IN EDUCATION AND AGING
PROFESSOR OF GERONTOLOGY AND PSYCHOLOGY
USC DAVIS SCHOOL OF GERONTOLOGY
UNIVERSITY OF SOUTHERN CALIFORNIA
LOS ANGELES, CA 90089

SCIENTIFIC REVIEW OFFICER

MOTEN, CARMEN, PHD, MPH
SCIENTIFIC REVIEW OFFICER
NATIONAL INSTITUTE ON AGING
NATIONAL INSTITUTES OF HEALTH
BETHESDA, MD 20892

EXTRAMURAL SUPPORT ASSISTANT

JIMENEZ, ARMILDA G.
EXTRAMURAL SUPPORT ASSISTANT
NATIONAL INSTITUTE ON AGING
NATIONAL INSTITUTES OF HEALTH
BETHESDA, MD 20892

* Temporary Member. For grant applications, temporary members may participate in the entire meeting or may review only selected applications as needed.

Consultants are required to absent themselves from the room during the review of any application if their presence would constitute or appear to constitute a conflict of interest.
